# Different glycosylation profiles of cystatin F alter the cytotoxic potential of natural killer cells

**DOI:** 10.1007/s00018-023-05041-x

**Published:** 2023-12-13

**Authors:** Emanuela Senjor, Martina Pirro, Urban Švajger, Mateja Prunk, Jerica Sabotič, Anahid Jewett, Paul J. Hensbergen, Milica Perišić Nanut, Janko Kos

**Affiliations:** 1 Department of Biotechnology, Jožef Stefan Institute, Ljubljana, Slovenia; 2 Faculty of Pharmacy, University of Ljubljana, Aškerčeva Cesta 7, 1000 Ljubljana, Slovenia; 3 Center for Proteomics and Metabolomics, Leiden University Medical Center, Leiden, The Netherlands; 4 Blood Transfusion Centre of Slovenia, Ljubljana, Slovenia; 5 Division of Oral Biology and Medicine, The Jane and Jerry Weintraub Center for Reconstructive Biotechnology, School of Dentistry, University of California Los Angeles, Los Angeles, USA; 6 The Jonsson Comprehensive Cancer Center, Los Angeles, USA

**Keywords:** Cystatin F, Immunosuppression, NK cells, N-Glycosylation

## Abstract

Cystatin F, a cysteine peptidase inhibitor, is a potent modulator of NK cytotoxicity. By inhibiting granule-mediated cytotoxicity pathway, cystatin F induces formation of non-functional NK cell stage, called split-anergy. We show that N-glycosylation determines the localization and cellular function of cystatin F. Cystatin F mostly exhibited high-mannose glycosylation in U-937 cells, both high-mannose and complex glycosylation in NK-92 and primary NKs, and predominantly complex glycosylation in super-charged NKs. Manipulating N-glycosylation with kifunensine increased high-mannose glycosylation of cystatin F and lysosome localisation, which decreased cathepsin C activity and reduced NK cytotoxicity. Mannose-6-phosphate could significantly reduce the internalization of extracellular cystatin F. By comparing NK cells with different cytotoxic potentials, we found that high-mannose cystatin F was strongly associated with lysosomes and cathepsin C in NK-92 cell line. In contrast, in highly cytotoxic super-charged NKs, cystatin F with complex glycosylation was associated with the secretory pathway and less prone to inhibit cathepsin C. Modulating glycosylation to alter cystatin F localisation could increase the cytotoxicity of NK cells, thereby enhancing their therapeutic potential for treating cancer patients.

**Graphical abstract:**

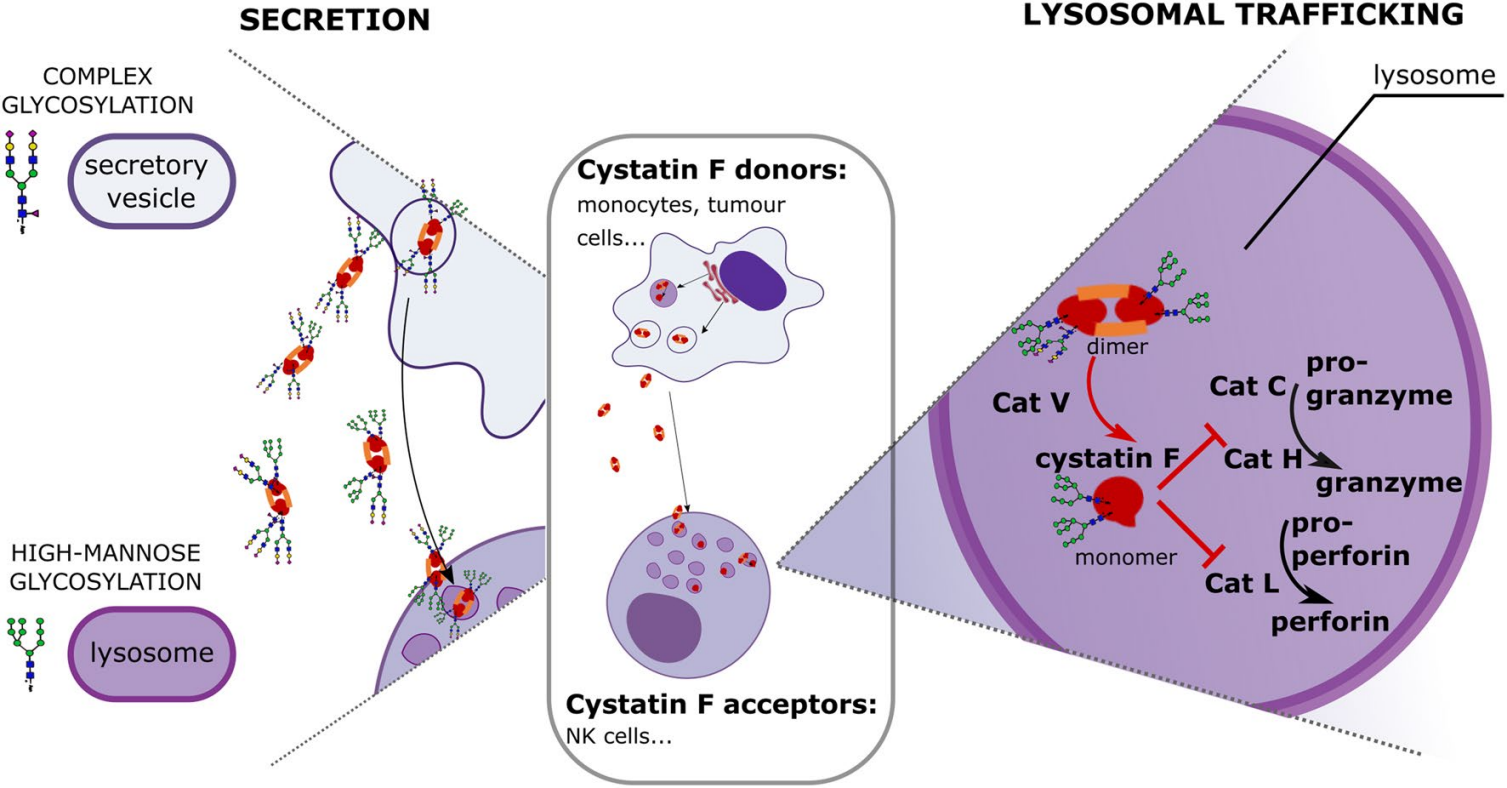

**Supplementary Information:**

The online version contains supplementary material available at 10.1007/s00018-023-05041-x.

## Introduction

Natural killer (NK) cells comprise 5–10% of peripheral-blood lymphocytes and have many functions, including anti-cancer, anti-viral, and anti-graft-versus-host disease action. Furthermore, they can eliminate cancer stem cells without prior antigen sensitization; thus NK cell-based immunotherapy has emerged as a promising therapeutic approach for solid tumors and hematological malignancies [1]. However, changes in their effector function after transfer to cancer patients, such as loss of cytotoxic potential and increased proinflammatory cytokine secretion, still hinder their effective use in cancer immunotherapy [2–8].

One of the main mechanisms that NK cells deploy for killing target cells is granule-mediated cytotoxicity, i.e., releasing cytotoxic granules into immune synapses. Such granules contain perforin and granzymes, which are effector molecules that induce apoptosis of target cells [9]. Lysosomal cysteine peptidases, cathepsins C and H, are important for granzyme activation, whereas, cathepsin L is involved in perforin activation. Their activity is regulated by endogenous inhibitors, cystatins [10, 11], including cystatin F, which is specifically expressed in immune cells [12], such as monocytes, NK cells [7], cytotoxic T cells (CTLs) [13], and CD4 + T cells [14]. In tumor microenvironment NK cells can exist in four functional states. Their transition from the fully functional state to split-anergy state is associated with decreased cytotoxicity, whereas their progress to a dysfunctional state is accelerated with further interactions with the immunosuppressive factors [15]. Cystatin F levels are higher in NK cells with impaired effector function, in which it inhibits the activities of cathepsin C and H and consequently reduces the levels of active granzymes [7]. Extracellular cystatin F can be internalised and trafficked to endo/lysosomal vesicles and can thus reduce the granule-mediated cytotoxicity of NK-92, primary NK cells [16], and CTLs [13, 17].

Protein glycosylation enables optimal protein folding, stability, activity, and trafficking and thus indirectly modulates the intra- and intercellular processes necessary for cellular homeostasis. Glycosylation machinery is template-free; however, cells have been shown to reproducibly attach glycans to specific protein sites via O- and N-glycosylation [18–20]. Many cell surface and secreted molecules involved in innate and adaptive immune responses are N-linked glycoproteins. Changes in their glycosylation patterns may lead to significant alterations in immune pathway signaling. Despite increasing knowledge on the regulatory role of glycosylation in immune responses, the molecular mechanisms triggering these processes remain unknown [21].

N-Glycosylation of proteins begins in the secretory pathway in the endoplasmic reticulum and Golgi apparatus by the transfer of glycan precursors (Glc3Man9GlcNAc2) to Asn residues (Asn-X-Ser/Thr/Cys, where X cannot be Pro). Further sequential trimming and elongation by specific glycosidases and glycosyltransferases result in high-mannose N-glycoproteins (Man5-9GlcNAc2). These glycoproteins may escape additional modifications or are further processed to a hybrid type, with partially exposed mannose, or finally to complex *N*-glycans [22]. Mannose glycans can be modified to mannose-6-phosphate (M6P), which directs protein transport to the endo-lysosomal system [21]. In general, secreted glycoproteins in mammalian cells are usually more processed (i.e., exhibit hybrid/complex glycosylation) compared to intracellular proteins [23]. In normally differentiated and viable cells, high-mannose glycans are predominantly intracellular signatures used for lysosomal targeting [24]. Therefore, human cells can generate microheterogeneous glycoproteins that can be localized in different subcellular compartments [23]. In addition, sites on the same protein can reproducibly receive different types of glycans, depending on the availability of activated sugar donors, relative enzyme abundance, competition among multiple enzymes for the same glycan substrates, and differential site accessibility for glycosylation processing [18, 21, 23].

Cystatin F has two canonical N-glycosylation sites (Asn62 and Asn115) [25] and is synthesized as a dimer, bound by disulphide bonds [26]. To inhibit cathepsin C, H, and L, it is transferred to endo/lysosomes by Asn62 N-linked glycosylation. In the lysosomes cystatin F is converted to a monomer by proteolytic cleavage of the 15-amino-acid N-terminal peptide by cathepsin V [27–29]. It was proposed that glycosylation stabilizes the dimeric form [30]. Furthermore, similar to other type II cystatins, cystatin F is secreted in its dimeric form into the extracellular space. Asn115 glycosylation mediates the secretion of cystatin F [28]. From extracellular space cystatin F can be internalized into bystander cells, transferred to endo/lysosomes, activated by conversion to monomeric form, enabling in trans inhibition of its targets [28, 31]. It has already been suggested that the glycosylation status of cystatin F is important for its intracellular function, as mutating the glycosylation site Asn62 prevented endo/lysosomal trafficking and enhanced secretion of cystatin F [16, 28].

The aim of this study was to analyse the glycosylation status of cystatin F in different types of NK cells and its association with cell function. We demonstrated the importance of high-mannose N-glycosylation for the localization of cystatin F in endo/lysosomal vesicles and for impaired cytotoxic function of NK cells. By manipulating the glycosylation machinery, we could redirect cystatin F trafficking and improve the cytotoxic potential of NK cells for cancer immunotherapy.

## Results

### Glycosylation of cystatin F differs between different cell types

**Fig. 1:**
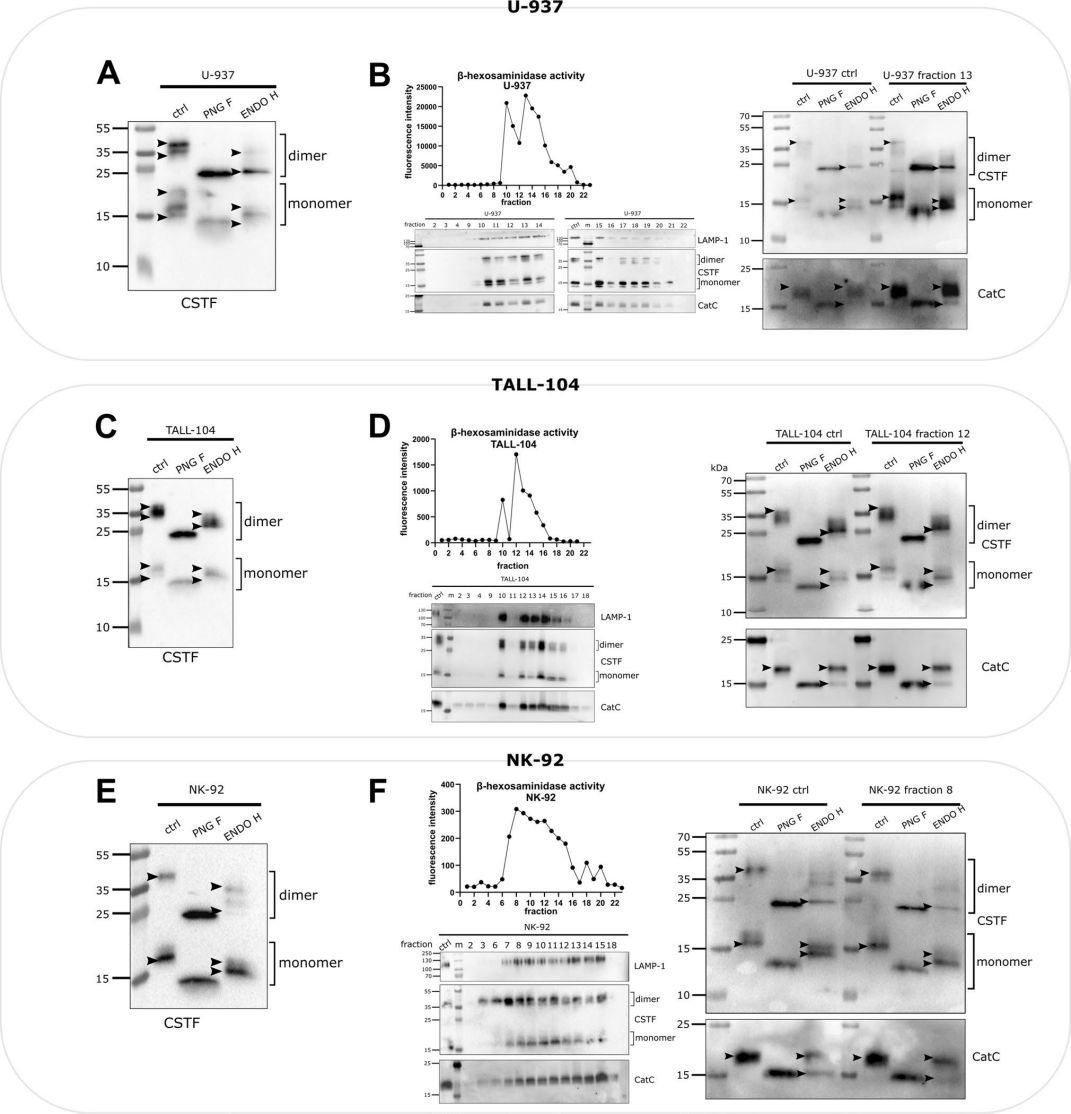
Glycosylation of cystatin F differs among cell types. Western blot of cystatin F (CSTF) in control and PNG F- and ENDO H-treated cell lysates of U-937 (**A**), TALL-104 (**C**), and NK-92 (**E**) cells. Arrowheads point to the highest and lowest bands for easier comparison between lanes. Activity of β-hexosaminidase in fractions obtained after ultracentrifugation of U-937 (**B**), TALL-104 (**D**), and NK-92 (**F**) cell lysates (top left). Western blot of LAMP-1, CSTF, and cathepsin C (CatC) in fractions obtained after ultracentrifugation of U-937 (**B**), TALL-104 (**D**), and NK-92 (**F**) cell lysates (bottom left). Western blot of CSTF and CatC expression in control and PNG F- and ENDO H-treated post-nuclear and lysosomal cell lysate fractions obtained after ultracentrifugation of U-937 (**B**), TALL-104 (**D**), and NK-92 (**F**) cells (right)

The glycosylation profile of intracellular cystatin F was analysed in three immune cell lines: promonocyte line U-937, NK-92, a model cell line for NK cells, and TALL-104, a model cell line for cytotoxic T cells [32]. For this purpose, liquid chromatography with tandem mass spectrometry (LC–MS/MS) analyses of tryptic digests were performed, followed by Byonic searches. This showed that Asn62 was the predominant glycosylated site of cystatin F in all three cell lines (Table 1). In addition, M6P modification was detected only at the glycosylation site Asn115 in all cell lines. However, the overall glycosylation profile of cystatin F differed between different cell types, with mainly high-mannose glycosylation in U-937 cells, both complex and high-mannose glycosylation in NK-92 cells, and a paucimannose type of glycosylation in TALL-104 cells (raw data in Supplemental Table S1). To corroborate these findings, cystatin F glycosylation profiles were evaluated by western blot analysis after treatment of whole cell lysates with Peptide-N-Glycosidase F (PNG F), which removes almost all N-linked glycans from the protein body, or Endoglycosidase H (ENDO H), which removes only high-mannose glycans [33]. Comparison of migration and band distribution between PNG F and ENDO H modified samples can give us an estimation of the susceptibility of *N*-glycans to ENDO H treatment. Western blot analysis results were similar to those of LC–MS/MS. Both cystatin F dimers and monomers in U-937 cells were modified with high-mannose glycosylation (Fig. 1A). Glycosylation of cystatin F was similar in lysosomes and whole cell lysates (Fig. 1B). The lysosomal fraction used for glycosylation analyses was obtained by ultracentrifugation in a Percoll/sucrose gradient and identified by high β-hexosaminidase activity and expression of lysosomal proteins, such as LAMP-1 and cathepsin C. TALL-104 cells had a similar glycosylation profile of cystatin F (Fig. 1C) as that in NK-92 cells (Fig. 1E): both showed high-mannose and complex glycosylation for monomeric and dimeric cystatin F. However, in contrast to NK-92 cells, TALL-104 cells did not exhibit different glycosylation of cystatin F between whole cell lysates and lysosomal fractions (Fig. 1D). In NK-92 cells, both monomeric and dimeric cystatin F in lysosomes exhibited more high-mannose glycosylation than in whole cell lysates (Fig. 1F). In addition, cathepsin C glycosylation did not differ between the lysosomal fractions and whole cell samples in any of the cell lines. Another promyelocytic cell line, HL-60, had a similar cystatin F glycosylation profile as U-937, with high-mannose-glycosylated cystatin F being predominant, whereas TALL-104 cells exhibited a similar glycosylation profile to that of CD8 + T cells, with both high-mannose and complex glycosylated cystatin F (Fig. S1). To summarize, cystatin F is heterogeneously glycosylated in different cell types, in monocytic cell lines, like U-937, cystatin F is modified mainly with high-mannose glycosylation, whereas in cytotoxic cell lines, like NK-92 and TALL-104, cystatin F is present in both high-mannose and complex glycosylated forms.

**Table 1:** The type of glycosylation of cystatin F in U-937, NK-92, and TALL-104 cells detected by LC–MS/MS analysis

	Glycosylation site	U-937	NK-92	TALL-104
DIMER	Asn62 (predominantly glycosylated)	High-mannose	High-mannose + complex	Paucimannose + complex
	Asn115	High-mannose M6P	High-mannose M6P	Paucimannose M6P
MONOMER	Asn62 (predominantly glycosylated)	High-mannose	High-mannose + complex	Paucimannose + complex
	Asn115	High-mannose M6P	High-mannose M6P	Paucimannose M6P

Only glycopeptides with a Byonic score above 200 were included in the analysis

### The intracellular localization of cystatin F can be modified with N-glycosylation inhibitors

**Fig. 2:**
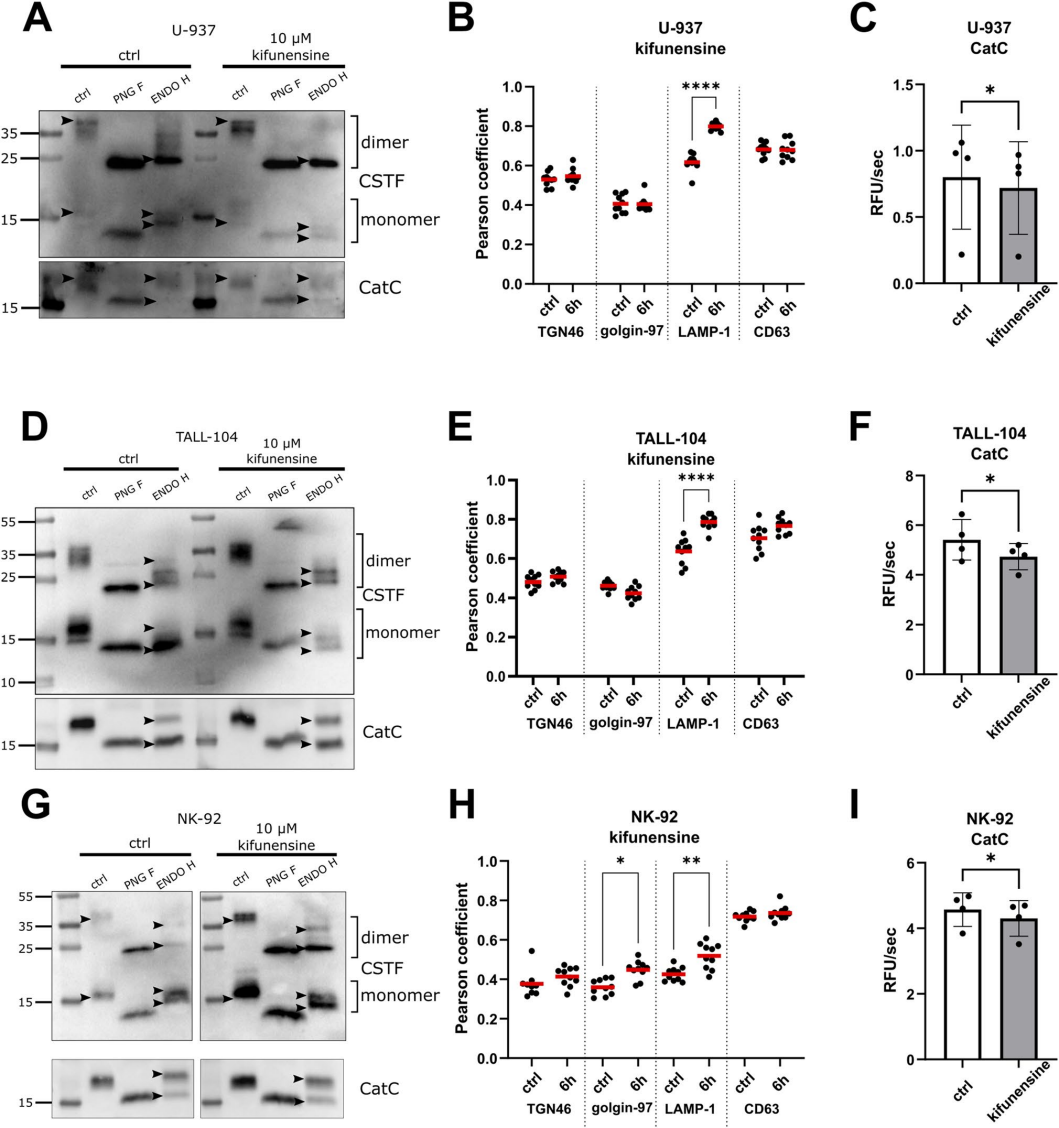
Modulating cystatin F glycosylation with kifunensine alters cystatin F localization and cathepsin C activity. Western blot of cystatin F (CSTF) and cathepsin C (CatC) expression in cell lysates of kifunensine-treated (10 µM, 18 h) U-937 (**A**), TALL-104 (**D**), and NK-92 (**G**) cells. Arrowheads point to the highest and lowest bands for easier comparison between lanes. Pearson correlation coefficients of CSTF colocalization with TGN46, golgin-97, LAMP-1, and CD63 in kifunensine-treated (10 µM, 6 h) U-937 (**B**), TALL-104 (**E**), and NK-92 (**H**) cells. Pearson coefficients were determined for 10 fields of view for each sample at × 63 magnification. The activity of CatC in kifunensine-treated (10 µM, 18 h) U-937 (*n* = 4) (**C**), TALL-104 (*n* = 4) (**F**), and NK-92 (*n* = 4) (**I**) cells. Control, untreated cells are shown with white bars and treated samples are shown in grey bars. Asterisks show statistical significance: *p < 0.05, **p < 0.01, ****p < 0.0001

We tested the effects of modulating N-glycosylation on the localization and function of cystatin F in U-937, TALL-104, and NK-92 cells. We have cultured the cells in the presence of N-glycosylation inhibitors tunicamycin and kifunensine. Tunicamycin completely prevents *N*-glycan formation by inhibiting GlcNAc phosphotransferase and thus blocks the transfer of *N*-acetylglucosamine-1-phosphate from UDP-*N*-acetylglucosamine to dolichol phosphate [22]. In addition, we tested kifunensine, a mannosidase I inhibitor that stops N-glycosylation of glycoproteins at the stage of high-mannose glycans [34]. Treatment of U-937 (Fig. S2A), TALL-104 (Fig. S2D), and NK-92 cells (Fig. S2G) with tunicamycin did not affect the glycosylation of monomeric cystatin F already formed and localized to the lysosomes of cells. However, newly formed cystatin F was not glycosylated and was secreted from the cells (Fig. S3). Cystatin F colocalization with different subcellular markers was analysed with confocal microscopy. Images were then processed to determine Pearson coefficients of correlation of cystatin F with each localization marker. Lysosomal localization of cystatin F was decreased in tunicamycin-treated U-937 (Fig. S2B), and NK-92 (Fig. S2H) cells, which was associated with a significant increase in cathepsin C activity upon tunicamycin treatment observed in U-937 (Fig. S2C) and NK-92 (Fig. S2I) but not in TALL-104 (Fig. S2F) cells. Conversely, the glycosylation profile of cystatin F changed, with high-mannose glycosylation predominating after kifunensine treatment, as ENDO H-treated bands shifted in kifunensine-treated samples compared to controls. The effects of kifunensine treatment of U-937 (Fig. 2A), and NK-92 (Fig. 2G) cells can be more clearly observed in the dimers of cystatin F than in monomers, whereas the change might not be as clear for Tall-104 cells (Fig. 2D). However there is still a clear increase in lysosomal localization of cystatin F (Fig. 2B, E, H) and in turn decreased cathepsin C activity (Fig. 2C, F, I) in all three cell lines. Representative confocal images are shown in Fig. S4. Interestingly, western blot analysis of cathepsin C glycosylation in tunicamycin- or kifunensine- treated cells did not differ from controls in any cell line (Fig. S2A, D, G and Fig. 2A, D, G). In summary, cystatin F glycosylation and consequently its colocalization and interaction with its target cathepsin C can be manipulated with changed glycosylation. Nonglycosylated cystatin F is mainly secreted, whereas high-mannose glycosylated cystatin F is increasingly translocated to the endo-lysosomal vesicles, where it can regulate the activity of cathepsin C.

### Glycosylation of cystatin F differs between NK cells with different cytotoxic potentials

**Fig. 3:**
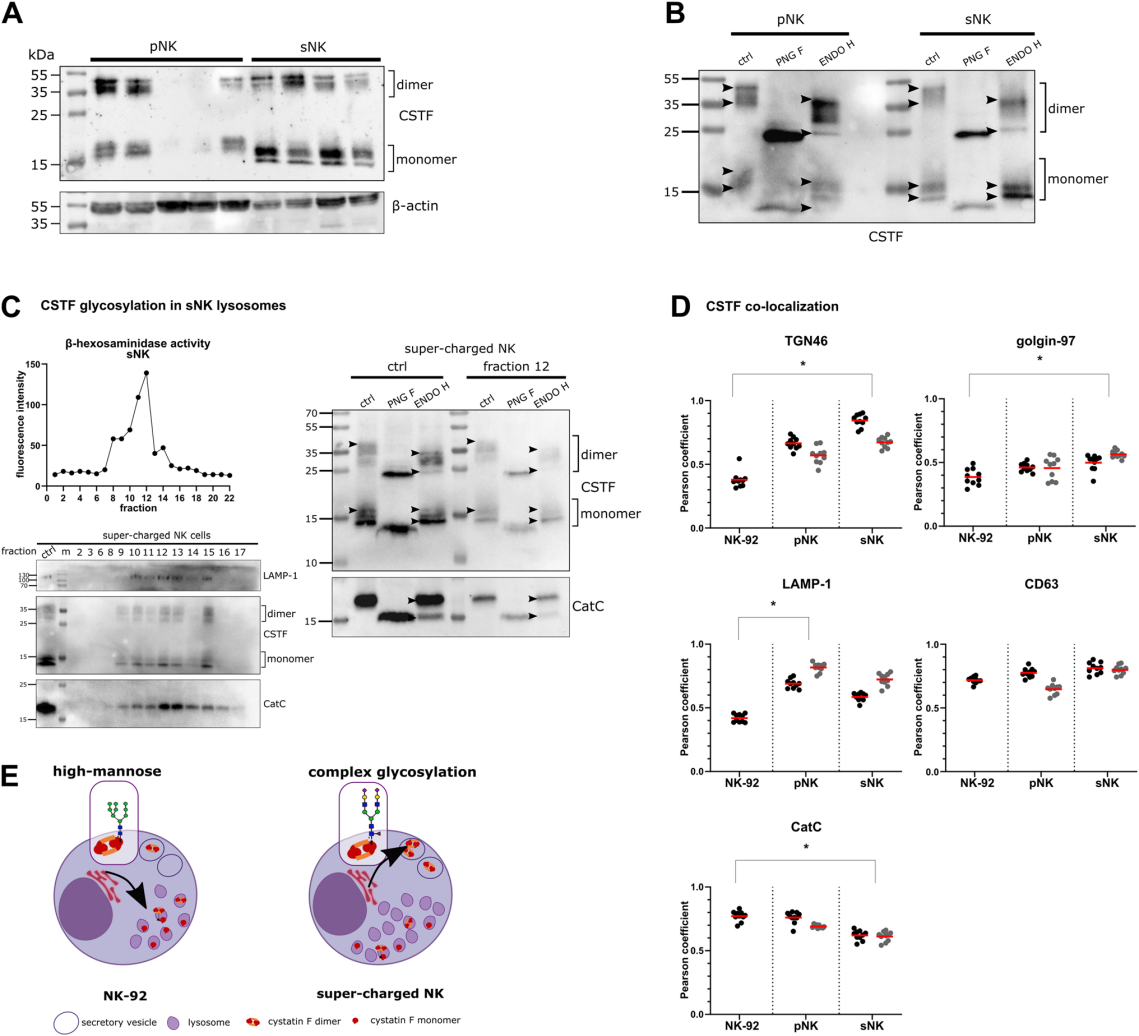
Cystatin F is differently glycosylated and thus differently localized in NK cells with different cytotoxic potentials. **A** Western blot of cystatin F (CSTF) expression in primary (*n* = 5) (pNK) and super-charged (*n* = 4) (sNK) cells. **B** Western blot of CSTF expression in control (ctrl), PNG F-treated, and ENDO H-treated pNK and sNK cell lysates derived from the same donor. **C** Activity of β-hexosaminidase in fractions obtained after ultracentrifugation of sNK cell lysate (upper left). Western blot of LAMP-1, CSTF, and cathepsin C (CatC) in fractions obtained after ultracentrifugation of sNK cell lysate (lower left). Western blot of CSTF and CatC expression in control, PNG F-treated, and ENDO H-treated post-nuclear and lysosomal cell lysate fractions obtained after ultracentrifugation of sNK cells (right). Arrowheads point to the highest and lowest bands for easier comparison between lanes. **D** Pearson coefficients of CSTF colocalization with TGN46, golgin-97, LAMP-1, CD63, and CatC in NK-92 (*n* = 1), pNK (*n* = 2), and sNK (*n* = 2) cells. Pearson coefficients were determined for ten fields of view for each sample under × 63 magnification. Cells derived from the same donor are shown in grey. Nested ANOVA was performed to determine the statistical significance of the differences between cell types, regardless of donor origin (*p < 0.05). **E** Graphical representation of the proposed pathways of cystatin F trafficking in NK-92 and super-charged NK cells

The glycosylation profile of cystatin F in super-charged (sNK) NK cells differs from that in NK-92 and primary (pNK) NK cells. Generated according to the protocol established by Jewett et al. [35], sNK cells are highly cytotoxic, have higher cytokine secretion ability and proliferation potential, and represent a potent new tool for adoptive cell therapy of cancer [35, 36]. The expression levels of cystatin F in cell lysates from pNK and sNK cells derived from different healthy donors varied between donors (Fig. 3A). However, the differences in the mobility of cystatin F remained consistent when comparing samples from pNK and sNK cells, regardless of donor origin (Fig. 3A). To examine whether differences in cystatin F mobility are a result of post-translational modifications, such as glycosylation, cell lysates were incubated with glycosidases. The resulting differences in mobility indicated that cystatin F from NK-92 (Fig. 1F) and pNK (Fig. 3B) cells have a similar glycosylation profile, composed of both complex and high-mannose glycans on both dimeric and monomeric forms. Cystatin F from sNK cells showed a different glycosylation profile in which dimers exhibited both high-mannose and complex glycosylation, whereas monomers exhibited mostly complex glycosylation (Fig. 3B). Cystatin F glycosylation was altered in lysosomal fractions compared to that in whole cell fractions in NK-92 cells (Fig. 1F), but not in sNK cells (Fig. 3C).

The intracellular localization of cystatin F differs in cells with different cytotoxic potentials. Analysis of cystatin F colocalization with markers of the synthesis/secretory pathway (TGN46, golgin-97), lysosomes (LAMP-1, CD63), and target peptidase cathepsin C (Fig. S5) was performed in NK-92, pNK, and sNK cells, which show gradually increasing cytotoxicity towards oral squamous carcinoma stem cells [7, 15]. In the least cytotoxic NK-92 cells, cystatin F strongly colocalized with cathepsin C and weakly with markers of the secretory pathway. In contrast, in sNK cells, cystatin F colocalized the most with TGN46 and golgin-97 and the least with cathepsin C compared to the other cells. This suggests that the super-charging expansion process causes changes in intracellular trafficking of cystatin F, as demonstrated by comparing the localization of cystatin F in pNK and sNK cells derived from the same donor (Fig. 3D, see also representative confocal images in Figs. S4 and S5). To sum up in NK-92 cells high-mannose glycosylated cystatin F localizes predominantly in the lysosomes, whereas in sNK cells cystatin F with more complex glycosylation is present in secretory granules (Fig. 3E).

### N-Glycosylation inhibitors alter cystatin F localization in NK cells

**Fig. 4:**
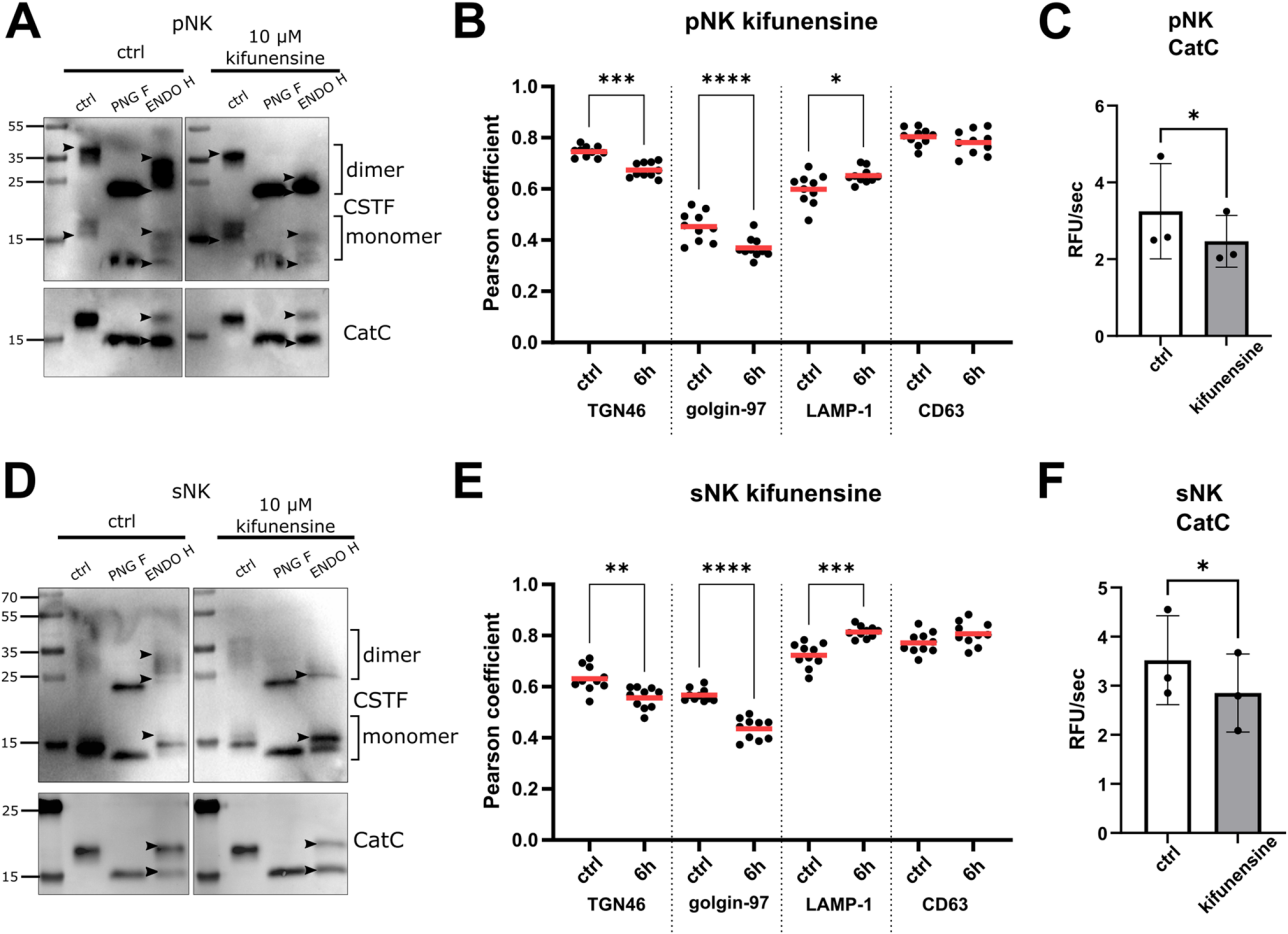
Kifunensine modifies cystatin F glycosylation and localization, resulting in altered cathepsin C activity. Western blot of cystatin F (CSTF) and cathepsin C (CatC) in cell lysates of primary NK (pNK) (**A**) and super-charged NK (sNK) cells (**D**) treated with kifunensine (10 µM for 18 h). Arrowheads point to the highest and lowest bands for easier comparison between lanes. Pearson correlation coefficients of CSTF colocalization with TGN46, golgin-97, LAMP-1, and CD63 in pNK (**B**) and sNK (**E**) cells treated with kifunensine (10 µM for 6 h). Pearson coefficients were determined for ten fields of view for each sample, under × 63 magnification. CatC activity in pNK (*n* = 3) (**C**) and sNK (*n* = 3) (**F**) cells in non-treated cells (white bars) and cells treated with kifunensine (10 µM for 18 h) (grey bars). *p < 0.05, **p < 0.01, ***p < 0.001, ****p < 0.0001

**Fig. 5:**
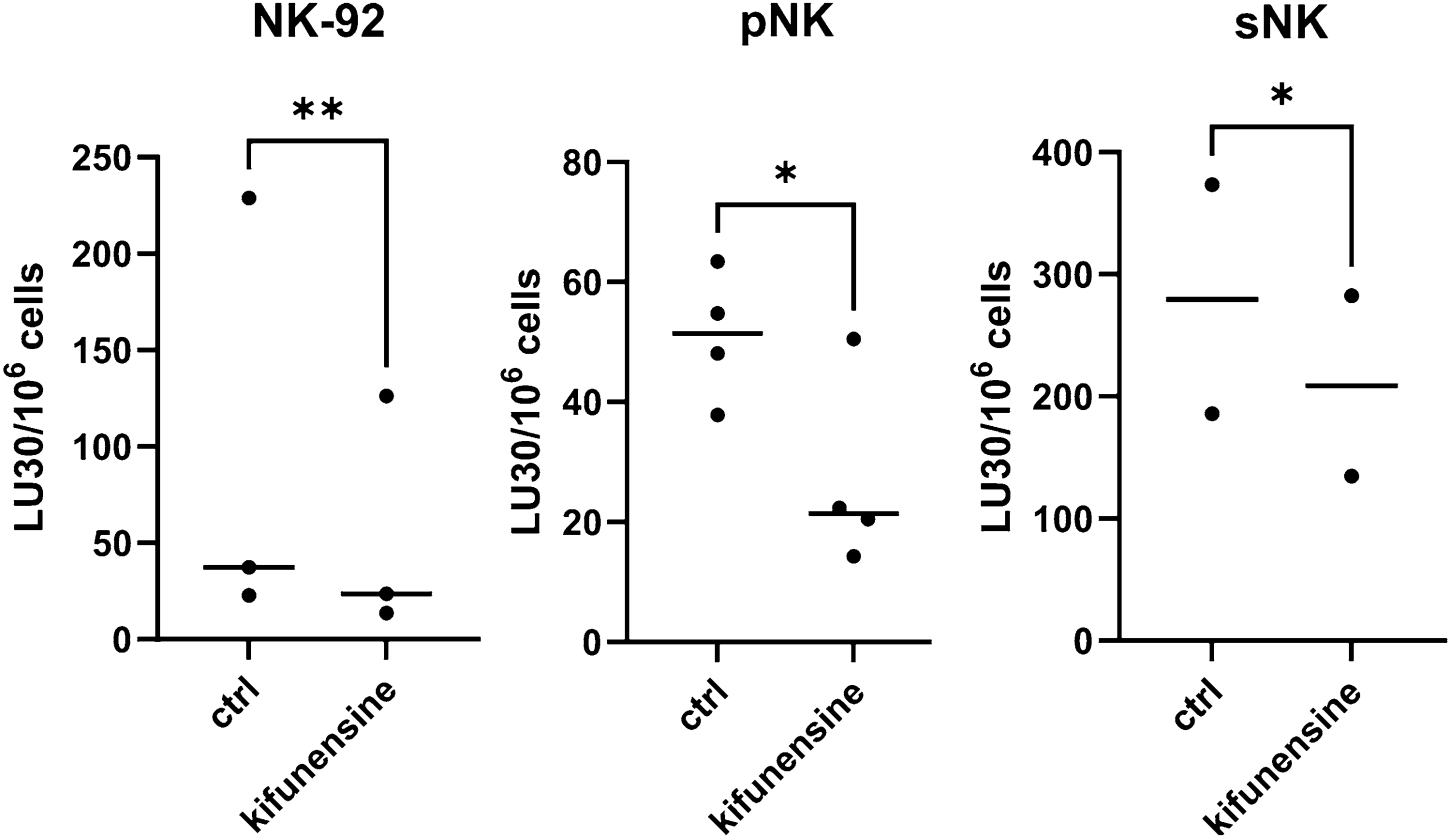
Kifunensine decreases cytotoxicity of NK-92 and primary NK cells. Calcein-AM release cytotoxicity assay of NK-92 (*n* = 3), primary NK (pNK, *n* = 4) and super-charged NK (sNK, *n* = 2) cells untreated (ctrl) or treated with kifunensine (20 µM for 6 h) prior to incubation with calcein-AM-labelled K-562 cells. LU 30/10^6^ cells were calculated using the inverse of the number of effectors needed to lyse 30% of the tumour cells × 100. *p < 0.05. % Cytotoxicity plots are found in Fig. S8

Both pNK and sNK cells were treated with kifunensine to increase the intracellular high-mannose glycosylated cystatin F fraction. Kifunensine shifted the glycosylation profile of cystatin F towards a more high-mannose profile but did not affect glycosylation of cathepsin C (Fig. 4A, D). We could observe greater differences in the glycosylation of the newly formed dimers in contrast to monomeric cystatin F forms. Altered cystatin F glycosylation was also reflected in altered cystatin F localization. After treating NK cells of different cytotoxic potentials with kifunensine, cystatin F associated more with the lysosomal marker LAMP-1 than with the synthesis/secretory pathway markers TGN46 and golgin-97 (Fig. 4B, E). Cathepsin C is the main target of cystatin F in lysosomes of cytotoxic T [13, 17, 37], cytotoxic CD4 + [14], and sNK cells (Fig. S6). Cathepsin C activity decreased with increased localization of cystatin F in lysosomes (Fig. 4C, F; see representative confocal images in Fig. S7). The decrease in cathepsin C activity was also reflected in the decreased cytotoxic potential of NK-92, primary NK cells and super-charged NK cells after kifunensine treatment (Figs. 5 and S8).

### Cystatin F reduces cytotoxicity while increasing IFNγ secretion from NK cells

**Fig. 6:**
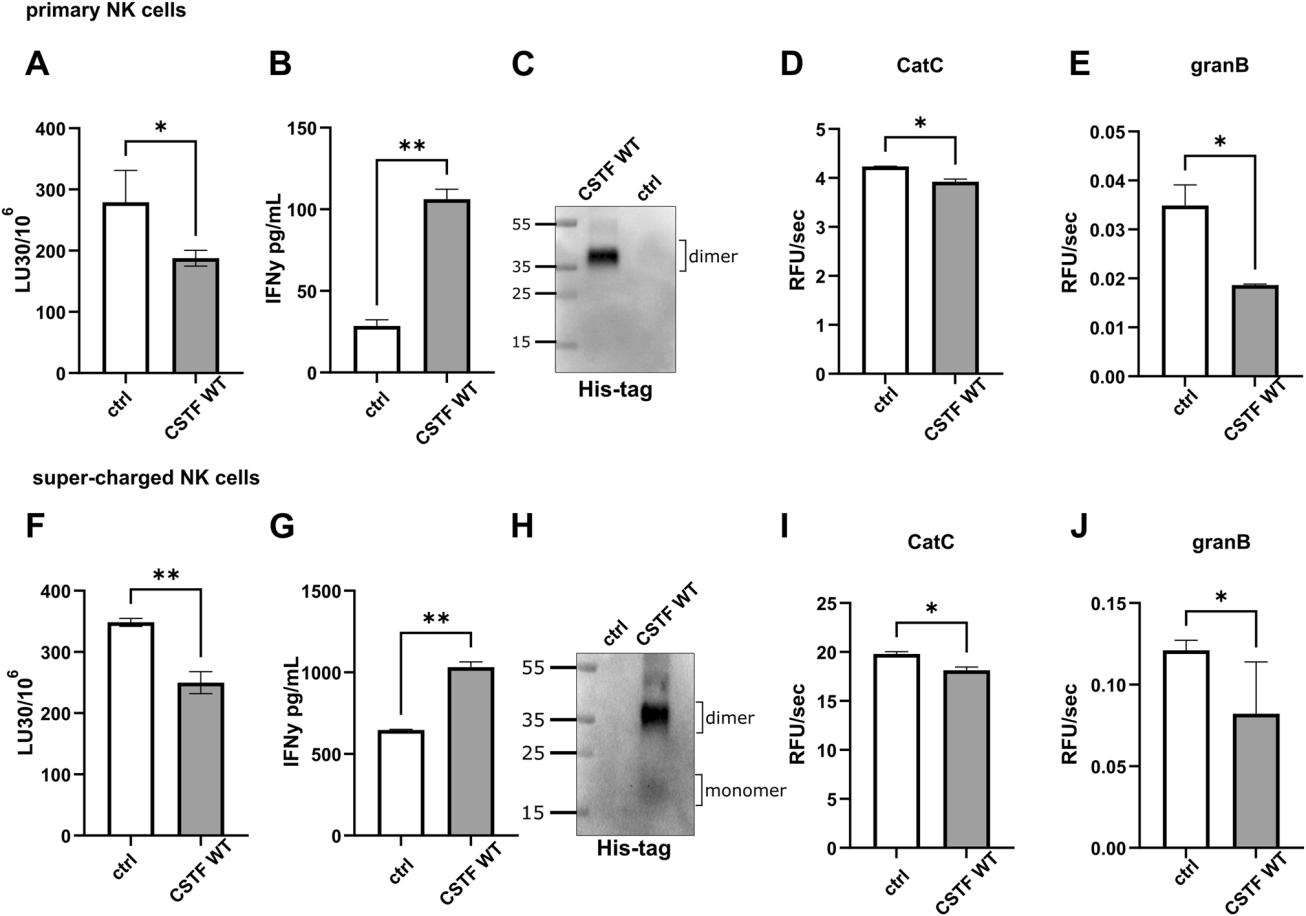
Cystatin F reduces cytotoxicity and increases IFNγ secretion from NK cells. Effects on cytotoxicity determined by flow cytometry of primary NK (**A**) and super-charged NK (**F**) cells after treatment with recombinant cystatin F (CSTF; 100 nM, 2 h). IFNγ secretion was measured by ELISA after treatment with recombinant CSTF (100 nM, 18 h) in primary NK (**B**) and super-charged NK (**G**). Western blot targeting the his-tag residue of recombinant CSTF in primary (**C**) and super-charged (**H**) NK cells was performed. Enzyme kinetics of cathepsin C (CatC) in cell lysates of primary (**D**) and super-charged (**I**) NK and granzyme B (granB) in cell lysates of primary (**E**) and super-charged (**J**) NK cells treated with recombinant CSTF (100 nM, 2 h). All assays were performed with primary and super-charged NK cells derived from same donor. *p < 0.05, **p < 0.01. Stain-free technology was used for western blot loading controls (Fig. S9A, B)

Cystatin F affects the function of both pNK and sNK cells. We have previously shown that treatment of IL-2-stimulated pNK and NK-92 cells with recombinant cystatin F significantly reduces their cytotoxicity [16]. To analyse whether cystatin F affects sNK cells in a similar way as pNK cells, we activated both cell types overnight with IL-2 and assessed their cytotoxicity and interferon γ (IFNγ) secretion after cystatin F treatment. Both pNK (Fig. 6A) and sNK (Fig. 6F) cells showed decreased cytotoxicity after cystatin F treatment (100 nM) and increased IFNγ secretion (Fig. 6B, G) compared to control cells (incubated with only IL-2). Western blotting confirmed the internalization of recombinant cystatin F into both cell types by targeting the his-tag on the recombinant protein to distinguish internalized cystatin F from endogenous cystatin F (Fig. 6C, H). Furthermore, internalization of cystatin F led to a decrease in cathepsin C and granzyme B activities in pNK (Fig. 6D, E) and sNK cells (Fig. 6I, J). Concurrently decreased NK cytotoxicity and increased IFNγ secretion are characteristics of split-anergy [4, 6, 8]. Similar results were obtained with NK cells derived from different donors (Fig. S9C, D, E).

### Mannose-6-phosphate reduces in trans cystatin F uptake

**Fig. 7:**
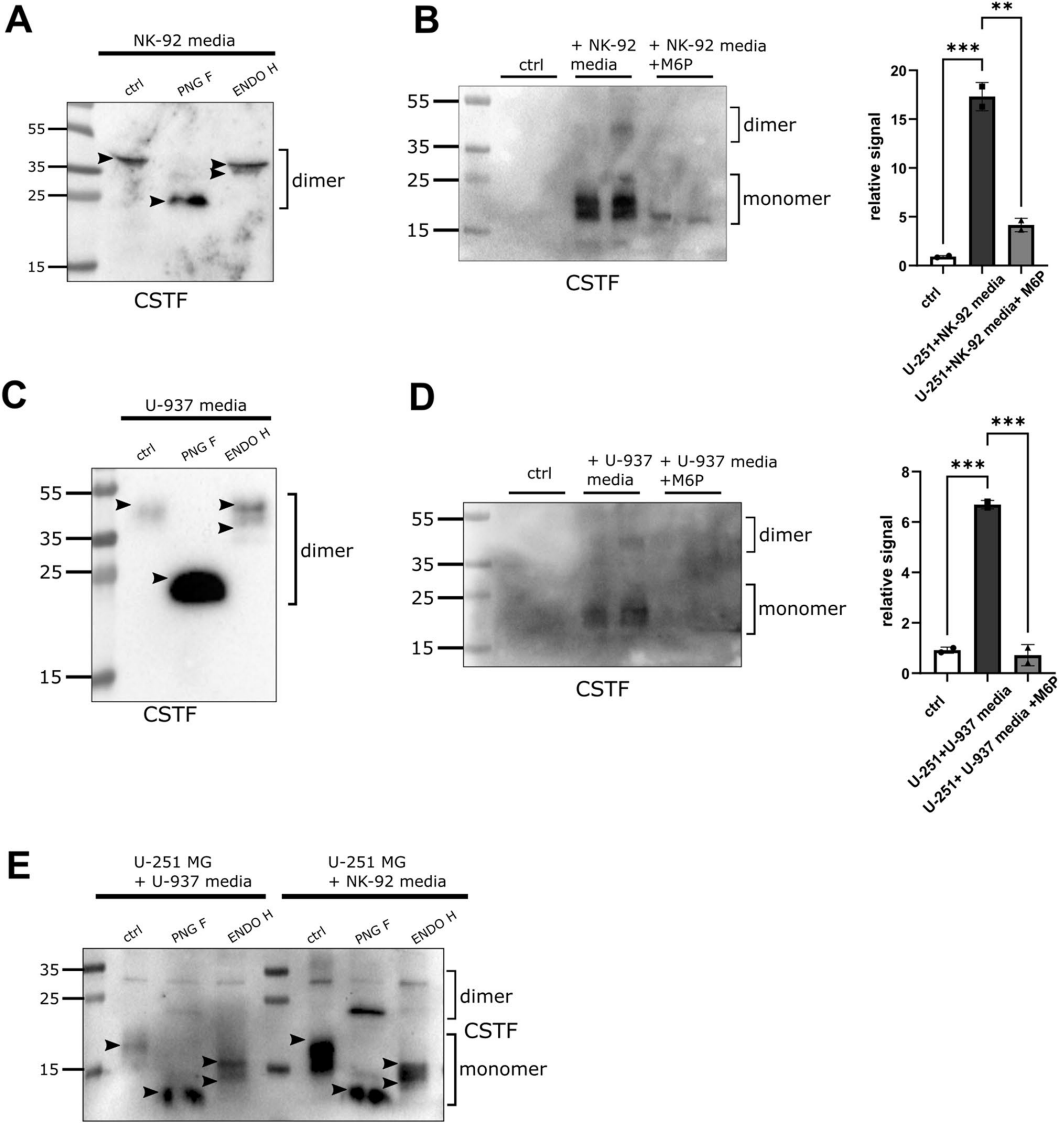
Internalization of secreted cystatin F into U-251 MG cells is reduced by M6P. Western blot of cystatin F (CSTF) expression in cell media secreted by NK-92 (**A**) and U-937 (**C**) cells. Western blot and signal quantification analysis of CSTF expression in control U-251 MG cells (white bars), cells cultured in conditioned NK-92 (**B**) and U-937 (**D**) media (black bars), or conditioned media with M6P (10 µM, 18h) (grey bars). **p < 0.01, ***p < 0.001. **E** Western blot of glycosylation of CSTF internalized into U-251 MG cells from U-937 or NK-92 conditioned media. Arrowheads point to the highest and lowest bands for easier comparison between lanes. Stain-free loading controls are shown in Fig. S10

Cystatin F secreted from NK-92 and U-937 cells mostly exhibited complex glycosylation (Fig. 7A, C). As previously shown, cystatin F can be internalized by bystander cells [17, 28]. Therefore, we analysed the glycosylation profile of internalized cystatin F. As a model, we used U-251 MG glioblastoma cells that do not express cystatin F. However, these cells stained positive for cystatin F after culturing in conditioned media from NK-92 (Fig. 7B) or U-937 cells (Fig. 7D). In accordance with previous data [28], we showed that M6P (10 µM) added to conditioned media of NK-92 or U-937 cells partially blocked cystatin F internalization into U-251 MG cells. A portion of the internalized cystatin F was sensitive to ENDO H treatment (Fig. 7E), suggesting both high-mannose and complex glycosylation of internalized cystatin F.

## Discussion

Cystatin F is an important modulator of the immune response, regulating the cytotoxic function of NK cells and CTLs by inhibiting cathepsin C, H, and L, the main activators of granzymes and perforin. Its function is controlled by specific expression in immune cells, a unique inhibitory profile, additional activation from the inactive dimeric form to monomeric form in the lysosomes, and trafficking pathways enabling either intra- or extracellular localization. In this study, we demonstrated that the glycosylation status affects cystatin F trafficking. Increasing high-mannose glycosylation caused cystatin F to be redirected to endo/lysosomal vesicles, impairing the cytotoxic function of NK cells. On the other hand, complex glycosylation, which is associated with the secretory pathway [23] was typical for cystatin F in sNK cells, which are known to exert high cytotoxicity and therapeutic effects [36].

We first examined the glycosylation profiles of recombinant and intracellular cystatin F in U-937, NK-92, and TALL-104 cell lines. Analysis of N-glycosylation with LC–MS/MS showed that Asn62 was predominantly glycosylated in all cell lines. However, the M6P modification was detected exclusively on Asn115. Detailed analysis of cystatin F N-glycosylation revealed differences among the analysed cells: high-mannose glycosylation in U-937 cells, both high-mannose and complex glycosylation in NK-92 cells, and paucimannose and complex glycosylation in TALL-104 cells. Recombinant cystatin F had paucimannose glycans (Fig. S11). It is known that glycosylation influences the localization of glycoproteins: high-mannose glycosylation is usually associated with lysosomes, whereas complex glycosylation is a signature of secreted proteins [24]. In addition, M6P modification enables protein targeting to lysosomes and internalization of extracellular proteins via M6P-dependent pathways [21]. The glycosylation profiles obtained by LC–MS/MS were consistent with the western blot analysis of PNG F- and ENDO H-treated cell lysates. By comparing band mobilities and distribution of the top and bottom bands corresponding to cystatin F, we can conclude whether complex (different mobility) or high-mannose glycosylation (same mobility) is predominant. Therefore, western blot analysis of PNG F- and ENDO H-treated cell lysates is a suitable method to gain a general overview of the glycosylation status of cystatin F. Nevertheless, we must take into account that western blotting cannot determine paucimannose glycosylation because ENDO H cannot cleave paucimannose glycans as effectively as high-mannose glycans [38, 39], resulting in less clear shifts in protein mobility. Therefore complementary methods such as LC–MS/MS are needed for data interpretations.

The paucimannose type of N-glycosylation, characterized by truncated mannose-terminating structures (Man1-3GlcNAc2Fuc0-1), was only recently discovered. At first, it was thought to exist only in plants and invertebrates, but was later also discovered in human glycoproteins in tumor tissues, cancer cell lines [40, 41], and neutrophilic granules [24, 42, 43]. In our case, the paucimannose type of N-glycosylation that we determined in TALL-104 cells by LC–MS/MS could be associated with a typical cancer origin or the presence of immune granules. In addition, by analysing glycosylation with western blotting, we showed that cystatin F in TALL-104 and CD8 + T cells has a similar glycosylation profile; however, the presence of paucimannose glycans in CD8 + T cells remain to be elucidated.

Different glycosylation patterns of the same protein in different immune cells have also been observed for other proteins. For example, the FCγRIIIa (CD16a) receptor exhibits high-mannose and complex glycosylation in NK cells but only complex glycosylation in monocytes, and this difference significantly affects ligand recognition [44–46]. In addition, many studies have shown differences in glycosylation between healthy and cancerous tissues. The most widely occurring cancer-associated changes in glycosylation are sialylation, fucosylation, *O*-glycan truncation, and *N*- and *O*-linked glycan branching [47]. Specific changes in glycan structures are associated with stemness, epithelial–mesenchymal transition, and escaping immunosurveillance [48, 49]. Taking into account that we used cancer cell lines to examine cystatin F glycosylation in this study, we demonstrated similar cystatin F glycosylation profiles between NK-92 and primary NK cells and between TALL-104 and CD8 + T cells.

Glycosylation machinery can be manipulated using various compounds. Kifunensine, for example, causes the production of high-mannose glycans. Multiple studies have shown that kifunensine can affect the localization of proteins by altering glycosylation, consequently influencing the effector function of immune cells [50–52], adhesion [53, 54], and aggregation [55] of tumour cells. In our study, kifunensine caused the production of high-mannose cystatin F, which increased the amount of cystatin F in lysosomes and decreased cathepsin C activity. We could observe greater influence of kifunensine on the dimeric cystatin F, where the protein migratory shift was more evident than in the monomeric cystatin F forms. Cystatin F is synthetized first in the dimeric form, which exist in differently glycosylated subsets which are triggered to different destinations—complexly glycosylated forms are more readily secreted, whereas high-mannose forms are transferred more readily to the lysosomes. Only in the lysosomes is cystatin F converted to monomer, therefore cystatin F monomers are more uniform in their glycosylation profiles compared to dimers. Due to that, we could observe clearer impact of the inhibition of N-glycosylation process with kifunensine. Surprisingly, kifunensine did not change the cathepsin C N-glycosylation pattern at the conditions used in this study. It is known that complexly glycosylated proteins have the slowest protein turnover [56]. Although cathepsin C exhibits both high-mannose and complex glycosylation, the ratio between the glycosylated forms might perhaps affect protein turnover, resulting in its decreased sensitivity to kifunensine compared to cystatin F.

In addition to kifunensine, we also tested tunicamycin, which completely prevents N-glycosylation. Tunicamycin alters the localization of glycoproteins, some of which can be trapped in the cell cytoplasm instead of trafficked to cell membrane [57], others are accumulated in the perinuclear region of cells [58]. Moreover, their secretion and nuclear transport can be increased and decreased, respectively, as it was shown for legumain [59]. In addition, tunicamycin reduces the cytotoxic activity of CTLs [60]. In our study, tunicamycin-treated cells exhibited secretion of deglycosylated cystatin F, decreased lysosomal localization of cystatin F, and consequently increased cathepsin C activity. Similarly as with kifunensine treatment, we could observe greater effects of tunicamycin on cystatin F dimers than monomers. Nevertheless, N-glycosylation is important for many cell processes, suggesting that tunicamycin may disrupt protein synthesis and turnover due to the instability of non-glycosylated proteins [61]. This is supported by findings that tunicamycin treatment of human colorectal carcinoma cells increased the secretion of less active (and less stable) legumain, which had lost its ability to be autoactivated [59]. Therefore, we cannot conclude whether the changed localization of cystatin F after tunicamycin treatment is due to increased secretion of non-glycosylated cystatin F or the overall decreased synthesis of cystatin F.

NK cells represent a promising tool for cancer immunotherapy because they can eliminate cancer stem cells without prior antigen sensitization and are activated when the balance between activating and inhibiting receptor–ligand interactions is disturbed [1]. NK cells can kill tumor cells via various mechanisms, of which the granule-dependent mechanism is the most important [62]. Cytotoxic granules, released into the immune synapse, contain effector molecules (perforin and granzymes), which induce apoptosis of target cells [9]. The effector molecules must be activated by lysosomal cysteine peptidases (cathepsins), and cystatins regulate the activity of cathepsins [10, 11]. It has been shown that monomeric cystatin F negatively affects immune cell cytotoxicity. By inhibiting cathepsins C and H, cystatin F prevents the activation of granzymes and perforin and thus decreases anti-tumor immune responses [7, 13, 16, 17].

Differences in N-glycosylation have been observed between various cells of the immune system. For example, cell surface glycosylation differs during T-cell development and activation [63]. In this study, we demonstrated that the N-glycosylation profile of cystatin F differs between NK cells with different cytotoxic potentials. When comparing the cytotoxic efficacy of NK-92, pNK, and sNK cells towards conventional target cells, sNK and NK-92 cells are the most and least cytotoxic, respectively [7, 15]. In our study, cystatin F expressed by NK-92 and pNK cells exhibited both high-mannose and complex glycosylation, whereas cystatin F expressed by sNK cells exhibited mostly complex glycosylation, indicating that the expansion and super-charging of NK cells alters protein glycosylation and subsequently localization. In another study, we have analysed the proteome and transcriptome of pNK and sNK cells and determined considerable differences in different biological pathways, among them in the N-glycosylation machinery (in prep). These differences in glycosylation significantly influenced cystatin F localization. In NK-92 cells, cystatin F was present in lysosomes and colocalized with cathepsin C. However, in sNK cells, cystatin F was highly localized in the secretory pathway and exhibited the lowest colocalization with cathepsin C among the analysed cell types. Treating NK-92, primary and super-charged NK cells with kifunensine resulted in a more high-mannose glycosylation profile of cystatin F, correlating with increased cystatin F concentrations within lysosomes and decreased cathepsin C activity. Therefore, high-mannose glycosylation of cystatin F seems to be important for the lysosomal trafficking of cystatin F and inhibition of cathepsin C. Although kifunensine was shown to render target K-562 cells more susceptible to NK cell killing [51], treating NK-92, primary, and super-charged NK cells with kifunensine resulted in increased cystatin F levels in lysosomes, decreased cathepsin C activity, and decreased cytotoxicity towards K-562 cells. These data confirm, that the high-mannose glycosylation status of cystatin F is important for its lysosomal localization and suppression of cytotoxic effector function of immune cells. Moreover, the changed localization of cystatin F in sNK cells due to complex glycosylation could be one of the mechanisms enabling increased cytotoxicity of sNK cells.

Nevertheless, endogenously expressed cystatin F in NK cells is only partly responsible for the limited cytotoxic potential of NK cells. Because cystatin F can act in trans, its expression by other immune cells in the tumor microenvironment or tumor cells [31, 64–66] can decrease the cytotoxic potential of NK cells as well. Cystatin F is secreted into the cell microenvironment as a dimer and can be internalized by bystander cells via the M6PR pathway [67, 68], which can be blocked by M6P [28].

Both endogenous and internalized cystatin F induces so-called split-anergy of NK cells, characterized by lower cytotoxicity and increased cytokine secretion [7, 16, 17]. We have shown that extracellular cystatin F can indeed decrease cathepsin C activity and cell cytotoxicity, and increase IFNγ secretion in both pNK [16, 69] and sNK cells. Previous studies indicated that secreted glycoproteins are more processed (i.e., exhibit hybrid and complex glycosylation), although some high-mannose rich proteins can also be secreted [23, 70]. Comparably, extracellular cystatin F secreted from U-937 or NK-92 cells predominantly exhibited complex N-glycosylation in our study. Secreted cystatin F was internalized into the U-251 MG glioblastoma cell line, which does not express cystatin F. We have found that Asn115 is modified by M6P, which is in agreement with previous studies that showed that Asn115 glycosylation is important for the secretion and internalization of cystatin F [16, 28]. In our study, the addition of M6P to the cell media reduced the internalization of cystatin F into U-251 MG cells. However, cystatin F was also internalized into U-251 MG cells by an M6P-independent mechanism, as suggested by Colbert et al. [28]. We observed ENDO H-sensitive bands in samples of conditioned cell media (especially of U-937 cells), suggesting that both high-mannose and complex glycosylated cystatin F are secreted, but that the high-mannose type is more readily internalized. Accordingly, we observed some sensitivity to ENDO H digestion in the cystatin F internalized into U-251 MG cells. Similarly, secreted recombinant cystatin F produced in HEK293F cells showed paucimannose glycosylation in LC–MS/MS and was sensitive to ENDO H cleavage, suggesting the presence of high-mannose glycans (Fig. S11).

Throughout the study, we could observe that cystatin F forms are heterogeneously glycosylated. The effects of tunicamycin or kifunensine treatment are more evident on cystatin F dimers, which could be explained by the fact that the dimeric form is the one that goes through the N-glycosylation process and has two different destinations-either it is secreted or transferred to the endo/lysosomes. Therefore, the presence of the high-mannose glycosylated cystatin F in tumor microenvironment could be detrimental to the anti-tumor immune response, as this type of cystatin F can be internalized into cytotoxic immune cells decreasing their cytotoxicity.

We can conclude that cystatin F is an important regulator of NK cell cytotoxicity. The type of glycosylation is important in mediating the function of cystatin F, as it affects the localization of cystatin F. Cystatin F with high-mannose glycosylation is translocated to lysosomes, where it interacts with cathepsin C and decreases the cytotoxicity of NK cells, switching them to the anergic state. Whereas sNK cells are still susceptible to the detrimental effects of extracellular cystatin F from other cell sources, endogenous cystatin F in sNK cells with complex glycosylation is less prone to affect their cytotoxicity. Modulating the glycosylation of endogenous cystatin F in NK cells could increase their anti-tumor function, while inhibiting the internalization of extracellular cystatin F via M6P could prevent its in trans action and the effect of the tumor microenvironment on NK cell cytotoxicity. Future studies should be focused on translating the results to complex human tissue models, such as organoids and microfluidic devices, which have advantages over animal models, including humanized mice, where key aspects of glycosylation pathways and peptidase expression can be considerably different.

## Materials and methods

### Cell lines

U-937, HL-60, U-251 MG, and K-562 cells were cultured in RPMI 1640 (Lonza, Switzerland), 10% heat-inactivated foetal bovine serum (HI-FBS) (Gibco, Massachusetts, USA) and 1% penicillin/streptomycin (P/S) (Gibco). NK-92 cells were cultured in RPMI 1640, 12.5% HI-FBS, 12.5% heat-inactivated horse serum, 200 IU/mL IL-2 (cat. 4030758, Bachem, Switzerland), and 1% P/S. TALL-104 cells were cultured in RPMI 1640 (ATCC, Virginia, USA) supplemented with 20% HI-FBS and 150 IU/mL IL-2. All cell lines were obtained from ATCC, tested negative for mycoplasma contamination, and were cultured at 37 °C and 5% CO_2_.

### Primary NK and CD8 + T cell isolation

Buffy coats were obtained from healthy volunteers at the Blood Transfusion Centre of Slovenia according to institutional guidelines (Ethics Committee of the Republic of Slovenia approval no. 0120-279/2017-3) or at the University of California, Los Angeles (UCLA). Written informed consents were obtained from healthy donors as approved by the UCLA Institutional Review Board (IRB) (11-000781); all procedures were approved and all methods were carried out in accordance with UCLA-IRB guidelines and regulations. Peripheral blood mononuclear cells were collected after Ficoll-assisted gradient centrifugation at the Blood Transfusion Centre of Slovenia or at UCLA. NK cells were isolated using a magnetic NK cell isolation kit with negative selection (cat. 130-092-657, Miltenyi Biotec, Germany) or EasySep Human NK Cell Enrichment kit (cat. 19055, Stem Cell Technologies). CD8 + T cells were isolated using EasySep Human CD8 + Cell Enrichment kit (cat. 19053, Stem Cell Technologies, Canada). The purity of the isolated cells was checked with a flow cytometer (FACSCalibur, Becton Dickinson Immunocytometry System, New Jersey, USA) with anti-CD3 (cat. 300440, BioLegend, California, USA), anti-CD56 (cat. 130-113-312, Miltenyi Biotec), anti-CD16 (cat. 130-120-871, Miltenyi Biotec), CD3/CD16 + CD56 (cat. 319101, BioLegend), anti-CD8 (cat. 300908, BioLegend), and anti-CD4 (cat. 300508, BioLegend) antibodies. Flow Jo software (TreeStar, Oregon, USA) was used for analysis. Primary NK and CD8 + T cells were cultured in RPMI 1640 media supplemented with 8% HI-FBS, MEM non-essential amino acids (Gibco), sodium pyruvate (Gibco), 1% P/S, and 1000 IU/mL IL-2.

### Super-charged NK cell expansion

Isolated peripheral-blood mononuclear cells were incubated on tissue culture plates in MEM alpha media (Gibco), containing 10% heat-inactivated autoplasma for 1 h. Plates were then washed with PBS, and the adhered monocytes were differentiated into osteoclasts by treatment with 25 ng/mL macrophage colony stimulating factor (cat. 574806, Biolegend) and 25 ng/mL RANKL (cat. 31001, Peprotech, New Jersey, USA) every 3 days for 3 weeks in MEM alpha media with 10% autoplasma. Isolated NK cells were treated overnight with 1000 IU IL-2/mL and anti-CD16 antibodies (cat. 302033, BioLegend) and then cultured on the osteoclast feeder layer, secreting IL-15, IL-12, IL-18, and IFN-α and expressing NKG2D ligands [71], with the addition of a mixture of sonicated probiotic bacteria sAJ2 [35]. Culture was supplemented every 2–3 days with fresh media (RPMI 1640, 10% human AB serum (cat. H5667, Sigma, Missouri, USA), MEM non-essential amino acids, sodium pyruvate, and 1% P/S). and 500 IU/mL of IL-2.

### Cytotoxicity assays

#### CFSE/7-AAD staining

Effector cells (primary NK cells or super-charged NK cells) were prepared in selected effector-to-target ratios, the highest being 5:1, with 5–8 serial dilutions in 96-well U-bottom plates. Effector cells were left untreated or treated with 100 nM recombinant wild-type cystatin F for 2 h. CSTF WT was prepared as described previously [16]. Meanwhile, target K-562 cells were stained with carboxyfluorescein succinimidyl ester (CFSE) (cat. C34554, Invitrogen, Massachusetts, USA) for 20 min at 37 °C, washed with media, and added (*n* = 20,000) to effector cells. The plate was centrifuged at 200*g* for 1 min and incubated for 4 h at 37 °C. Each well was transferred to a flow tube. The cells were washed twice and resuspended in PBS. 7-Amino-actinomycin D (7-AAD) was added to each flow tube (cat. SML1633, Sigma). After incubation on ice for 10 min, the samples were run in an Attune NxT flow cytometer (Thermo Fisher Scientific, Massachusetts, USA). The results were analysed with FlowJo. Briefly, spontaneous lysis of target cells (wells that contained only target cells but no effector cells) was subtracted from the percentage of dead target cells (CFSE- and 7-AAD-positive cells) determined for each sample. Lytic units (LU) 30/10^6 ^cells were calculated using the inverse of the number of effector cells needed to lyse 30% of the target cells × 100.

#### Calcein-AM release assay

NK-92 cells were activated overnight with 1000 IU/mL IL-2, prepared at selected effector-to-target ratios, and treated with 20 µM kifunensine for 6 h. K-562 target cells were labelled with 15 µM calcein-AM (cat. 17783, Sigma) stain in serum-free media for 30 min. The cells were washed and added (*n* = 5000) to effector cells. The plate was centrifuged at 200*g* for 1 min and incubated for 3 h at 37 °C with 5% CO_2_. After incubation, the plate was centrifuged at 700*g* for 5 min, and 50 μL of supernatant was transferred to a new microtiter plate for fluorescence measurements. Released calcein-AM was measured using the Tecan M1000 microplate reader at 496 nm excitation and 516 nm emission. The percentage of cytotoxicity was calculated as: 100 × (test release − spontaneous release)/(total release − spontaneous release). Spontaneous release of calcein-AM was measured in wells containing 100 μL of NK-92 culture media and 50 μL of target cells. For total release, 2% Triton X-100 was added to the NK-92 culture media to achieve target cell lysis. LU were calculated using the inverse of the number of effector cells needed to lyse 30% of the target cells × 100.

### ELISA

The ELISA Max Standard Set Human IFNγ kit (cat. 430104, BioLegend) was used to measure IFNγ in NK cell supernatants. Briefly, capture antibodies were incubated on Nunc Immuno module Maxisorp strips (Thermo Scientific) overnight at 4 °C. The strips were washed with 0.05% Tween-20 PBS and blocked with assay diluent (1% BSA/PBS) for 1 h at room temperature. After washing, the standards and samples were added to the wells. Cell media was used to prepare standard and sample dilutions. After 2 h incubation at room temperature, the strips were washed, and the detection antibodies were added and incubated for 1 h. After washing, the avidin-horseradish peroxidase (HRP) solution was added. After 30 min of incubation, the strips were washed, and 3,3′,5,5′-Tetramethylbenzidine (TMB) substrate was added. The reaction was stopped with 2N H_2_SO_4_, and absorbance at 450 nm was measured with a microplate reader (Infinite M1000, Tecan, Switzerland).

### Western blot

Post-nuclear cell lysates were prepared in RIPA buffer containing protease inhibitors (Roche) and obtained after 30 min incubation on ice and 30 min centrifugation at 16,000*g*. Total protein concentration was measured using the DC protein assay (BioRad, California, USA). Non-reducing SDS PAGE was performed, and proteins were transferred to nitrocellulose membranes using the Trans-Blot Turbo system (BioRad). Membranes were blocked in 5% non-fat dry milk in PBS for 1 h and incubated overnight with primary antibodies. After incubation with HRP-conjugated secondary antibodies, the Clarity Max ECL substrate (BioRad) was used to visualize the bands in the ChemiDoc MP imaging system (BioRad). Blots were analysed using Image Lab software (BioRad).

The following antibodies were used: rabbit anti-His tag (cat. 12698S, Cell Signaling Technology, Massachusetts, USA), rabbit anti-cystatin F (cat. HPA040442, Sigma), mouse anti-β actin (cat. sc-517582, Santa Cruz Biotechnology, Texas, USA), mouse anti-LAMP-1 (cat. MAB 4800, R&D Systems), mouse anti-cathepsin C (cat. sc-74590, Santa Cruz Biotechnology), N135 sheep anti-cathepsin L [72], and mouse anti-GAPDH (cat. 10494-I-AP, Proteintech) primary antibodies, and anti-rabbit/mouse/sheep HRP (cat. 111-035-045, 115-035-068, 313-035-003, Jackson Immuno Research, Pennsylvania, USA), and anti-mouse StarBright 700 secondary antibodies (cat. 12004158, BioRad). Signal normalization analyses were performed using Stain-Free technology (BioRad).

### Enzyme kinetics

Cell lysates were prepared in 50 mM citrate buffer (pH 6.2 with 1% Triton X-100; for cathepsin C kinetics) or NP40 buffer (25 mM HEPES, 250 mM NaCl, 2.5 mM EDTA, pH 7.4, and 0.1% NP-40; for granzyme B kinetics). After 30 min incubation on ice and 30 min centrifugation at 16,000*g*, total protein concentration was measured using the DC protein assay (BioRad). Whole cell lysate proteins (10 µg) were activated in assay buffers for cathepsin C (25 mM MES, 50 mM NaCl, 5 mM DTT, pH 6; for 15 min at room temperature) and granzyme B (50 mM Tris, 100 mM NaCl, pH 7.4; 20 min at 37 °C). Then substrate was added: 70 µM Gly-Phe-AMC (Bachem) for cathepsin C or 50 µM AcIEPD (Bachem) for granzyme B. The Infinite M1000 microplate reader (Tecan) was used to measure the formation of fluorescent degradation products under continuous excitation at 370 and emission at 460 nm.

### Isolation of lysosomal fraction

Cells (100 × 10^6^) were lysed in lysis buffer (250 mM HEPES, 250 mM sucrose, pH 7.2) in a Dounce homogenizer on ice, until nearly all the cell membranes were disrupted (checked by Trypan Blue staining). After centrifugation for 10 min at 500*g*, the post-nuclear supernatant was separated on a 30% Percoll (GE Healthcare) and 250 mM sucrose gradient by ultracentrifugation for 70 min at 36,000*g* (Sorvall Lynx 4000 Thermo Scientific). Fractions were pelleted and resuspended in RIPA buffer. Protein concentration was measured using the DC protein assay (Biorad). Fractions containing lysosomes were determined by measuring β-hexosaminidase activity. Equal amounts of each fraction were added to 0.1 M sodium citrate, 0.2 M sodium hydrogen phosphate, pH 5, and 2 mM 4-methylumbelliferyl *N*-acetyl-*b*-d-glucosaminide. After 30 min incubation in the dark, the reaction was stopped with 0.2 M glycine, pH 10.3. The fluorescence was measured at 362 nm excitation and 448 nm emission with the Infinite M1000 microplate reader (Tecan). In addition, western blot was performed to determine the expressions of LAMP-1, cystatin F, and cathepsin C in each fraction.

### Immunocytochemistry

Coverslips were coated with poly-l-lysine (Sigma). After cells adhered to the coverslips, they were fixed with 4% paraformaldehyde/PBS for 20 min, washed with PBS, and permeabilized with 0.1% Triton X-100 for 10 min. The coverslips were then blocked with 3% BSA/PBS for 1 h at room temperature. Cells were then labelled with the following primary antibodies: rabbit anti-CSTF (Davids Biotechnologie, Germany), mouse anti-TGN46 (cat. SAB4200355, Sigma), mouse anti-golgin-97 (cat. sc-59820, Santa Cruz Biotechnology), mouse anti-LAMP1 (cat. MAB 4800, R&D Systems, Minnesota, USA), mouse anti-CD63 (cat. 10628D, Invitrogen), and mouse anti-cathepsin C (cat. Sc-74590, Santa Cruz Biotechnology) for 1 h at room temperature. After washing, coverslips were incubated with the secondary antibodies anti-rabbit 488 and anti-mouse 555 (cat. 4412S, 4409, Cell Signaling Technology) for 1 h at room temperature. Cells were then washed and mounted on slides overnight with ProLong Gold Antifade reagent with DAPI (Thermo Scientific). Slides were analysed using a LSM 710 confocal microscope (Carl Zeiss). ZEN 2.3 SP1 FP1 software and ImageJ software were used for image processing. Pearson correlation coefficients for ten field views per sample were determined with the JACoP2 plugin in ImageJ.

### Glycosylation inhibitors

Tunicamycin (1 µg/mL; Sigma) and kifunensine (10 µM; cat. 10009437, Cayman chemical company, Michigan, USA) were used to inhibit N-glycosylation or the formation of complex glycosylation, respectively. Cells were treated overnight (18 h) for western blot and enzyme kinetics and 6 h for immunocytochemistry.

### PNG F and ENDO H treatment

Lysates were prepared from cells treated with glycosylation inhibitors, and 10 µg of the lysates were treated with PNG F or ENDO H (cat. P0704L, P0702L, New England BioLabs, Massachusetts, USA) according to the manufacturer’s instructions. Briefly, lysates were denatured in 10% SDS at 100 °C for 10 min. Appropriate buffers supplied in the kit were added, and 1500 units of PNG F or ENDO H were added to the mixture. After 2 h of incubation at 37 °C, SDS PAGE and western blot were performed.

### Immunoprecipitation

Cells were lysed in 50 mM Tris, 100 mM NaCl, pH 7.4, 0.25% Triton X-100 with protease inhibitors and centrifuged at 4 °C and 16,000*g* for 30 min. Protein G Dynabeads (Invitrogen) were coated with 3 µg of rabbit anti-cystatin F (Davids Biotechnologie) or 3 μg of antibodies directed against lectin isolated from *Macrolepiota procera* (BioGenes GmbH) as a negative control. Dynabeads complexed with antibodies were incubated overnight at 4 °C with the cell lysate. Dynabead immunoprecipitates were washed with lysis buffer, resuspended in SDS loading buffer, and denatured by boiling at 100 °C for 10 min. The immunoprecipitates were analysed by western blotting.

### In-gel trypsin digestion and MS analysis

After cystatin F was pulled down by immunoprecipitation, the Dynabeads were magnetically removed, and SDS PAGE was performed (NuPAGE 4–12% Bis–Tris protein gels, Thermo Fisher Scientific). Gels were stained with SimplyBlue™ SafeStain (Invitrogen) for 3 h and washed with distilled water. For MS analysis, selected bands were cut from the gel.

In-gel trypsin digestion was performed using a Proteineer DP digestion robot (Bruker). Prior to digestion, proteins were first reduced and alkylated using dithiothreitol (DTT, 10 mM) and iodoacetamide (50 mM), respectively.

Tryptic peptides were extracted from the gel slices, lyophilized, dissolved in solvent A (95/3/0.1 water/acetonitril/formic acid (FA) v/v/v) and subsequently analysed by on‐line C18 nano-HPLC MS/MS with a system consisting of an Easy nLC 1000 gradient HPLC system (Thermo, Bremen, Germany), and a LUMOS mass spectrometer (Thermo). Samples were injected onto a homemade precolumn (100 μm × 15 mm; Reprosil-Pur C18-AQ 3 μm, Dr. Maisch, Ammerbuch, Germany) and eluted via a homemade analytical nano-HPLC column (15 cm × 50 μm; Reprosil-Pur C18-AQ 3 um). The gradient was run from 10 to 40% solvent B (20/80/0.1 water/acetonitrile/ FA v/v/v) in 30 min. The nano-HPLC column was drawn to a tip of ∼ 5 μm, and acted as the electrospray needle of the MS source. The LUMOS mass spectrometer was operated in data-dependent MS/MS (top-20 mode) with a normalised collision energy of 32% and recording of the MS2 spectrum in the Orbitrap. In the master scan (MS1), the resolution was 120,000 with a scan range *m*/*z* 400–2000. Dynamic exclusion after *n* = 1 with exclusion duration of 10 s was applied. For MS/MS, precursors were isolated with the quadrupole with an isolation width of 1.2 Th. The MS2 scan resolution was 30,000. During acquisition a ProductIonTrigger was set on the HexNAc oxonium ion at *m*/*z* 204.087. Upon detection of the oxonium ion, three additional data-dependent MS2 scans of the same precursor were executed with HCD normalised collision energies 32, 37 and 41%, respectively, at an AGC target of 500,000 (instead of 50,000 for the initial round of MS/MS) with a maximum fill time of 200 ms. In addition, a CID spectrum of the same precursor was recorded at a collision energy of 35%.

#### Data analysis

For (glyco)peptide identification, MS/MS spectra were searched against the human database (20,205 sequences, downloaded from Uniprot on April 8, 2020) with Byonic (Protein Metrics, version 3.10.10, California, USA). Precursor mass tolerance was set at 10 ppm and fragment tolerance at 20 ppm. Cleavage C-terminal of K and R was selected (fully specific) and a maximum of two missed cleavages was allowed. Fixed modification was carbamidomethyl (Cys) while oxidation of methionine was set as a variable modification. For glycopeptide assignment “*N*-glycan 309 mammalian no sodium” and “*N*-glycan Mannose-6-phosphate 20” modification lists were used. Only glycopeptides with a Byonic score > 200 were further selected.

### Cystatin F internalization from conditioned media

U-251 MG cells were plated on a 6-well plate and left to adhere overnight. U-937 or NK-92 conditioned media was prepared by culturing the cells at 1 × 10^6^/mL overnight (18 h). Cell suspension was centrifuged at 2000 rpm for 5 min to obtain conditioned cell media. U-251 MG cell media was removed and replaced with either 1 mL of control media, 1 mL conditioned media, or 1 mL conditioned media with 10 µM D-mannose-6-phosphate sodium salt (M6P) (Sigma). U-251 MG cell lysates were prepared in RIPA buffer after 18 h of treatment.

### Statistical analysis

Graphpad prism 9 software was used to perform statistical analysis with the *t* test or ANOVA as required. Values are presented as mean ± SD, and statistical significance was set at *p* < 0.05.

### Supplementary Information

Below is the link to the electronic supplementary material.

Supplementary file1 (XLSX 907 kb)

Supplementary file2 (DOCX 32903 kb)

## Data Availability

Data are available on reasonable request.
