# Supplementary material for: Different glycosylation profiles of cystatin F alter the cytotoxic potential of natural killer cells
Source: Cell Mol Life Sci. 2023 Dec 13;81(1):8. doi: 10.1007/s00018-023-05041-x (PMC10719177; doi:10.1007/s00018-023-05041-x)
Supplement: Supplementary file 2 — Supplementary file2 (DOCX 32903 kb) [file 18_2023_5041_MOESM2_ESM.docx]

DIFFERENCES IN GLYCOSYLATION PROFILES OF CYSTATIN F ALTER THE CYTOTOXIC POTENTIAL OF NATURAL KILLER CELLS

Emanuela Senjor^1,2^, Martina Pirro^3^, Urban Švajger^4^, Mateja Prunk^1^, Jerica Sabotič^1^, Anahid Jewett^5,6^, Paul J Hensbergen^3^, Milica Perišić Nanut^1^, Janko Kos^1,2^

^1^Department of Biotechnology, Jožef Stefan Institute, Ljubljana, Slovenia

^2^University of Ljubljana, Faculty of Pharmacy, Ljubljana, Slovenia

^3^Center for Proteomics and Metabolomics, Leiden University Medical Center, Leiden, The Netherlands

^4^Blood Transfusion Centre of Slovenia, Ljubljana, Slovenia

^5^Division of Oral Biology and Medicine, The Jane and Jerry Weintraub Center for Reconstructive Biotechnology, Department of Dentistry, University of California Los Angeles (UCLA), Los Angeles, USA

^6^The Jonsson Comprehensive Cancer Center, Los Angeles, USA

Supplement Materials

**
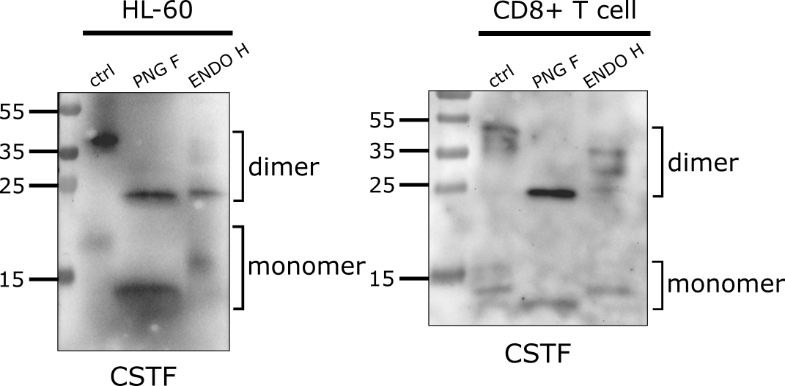
**

**Figure S1:** Western blot analysis of CST glycosylation in HL-60 (left) and CD8+ T cells (right).


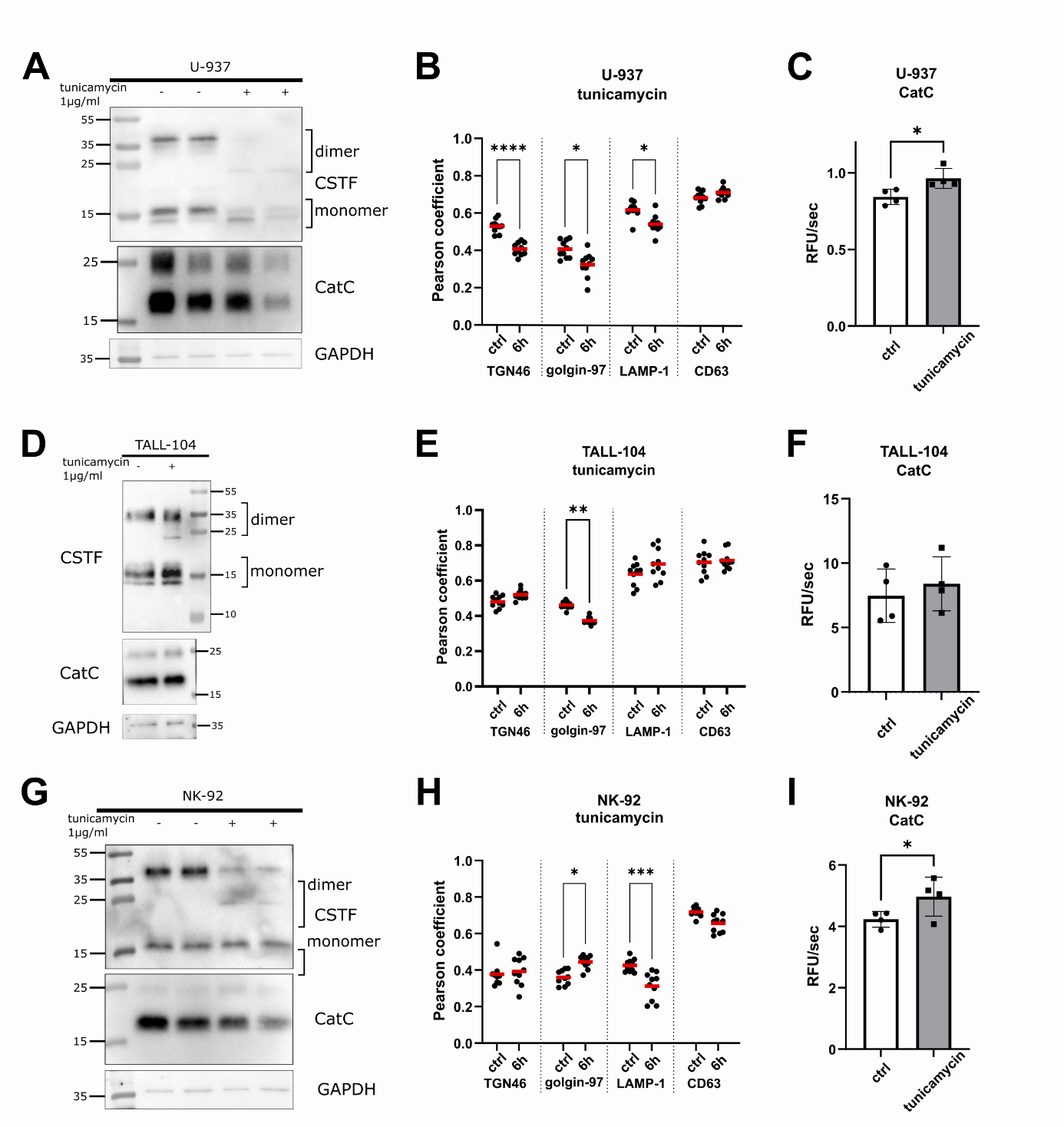


**Fig. S2: Modulating cystatin F glycosylation with tunicamycin alters cystatin F localization and cathepsin C activity**

Western blot of cystatin F (CSTF) and cathepsin C (CatC) expression in cell lysates of tunicamycin-treated (1 µg/mL, 18 h) U-937 (A), TALL-104 (D), and NK-92 (G) cells. Pearson correlation coefficients of CSTF colocalization with TGN46, golgin-97, LAMP-1, and CD63 in tunicamycin-treated (1 µg/mL, 6 h) U-937 (B), TALL-104 (E), and NK-92 (H) cells. Pearson coefficients were determined for 10 fields of view for each sample at 63× magnification. The activity of CatC in tunicamycin-treated (1 µg/mL, 18 h) U-937 (n=4) (C), TALL-104 (n=4) (F), and NK-92 (n=4) (I) cells. Control, untreated cells are shown with white bars, and treated samples are shown as grey bars. Asterisks show statistical significance: * p<0.05, ** p<0.01, ***p<0.001, **** p<0.0001.


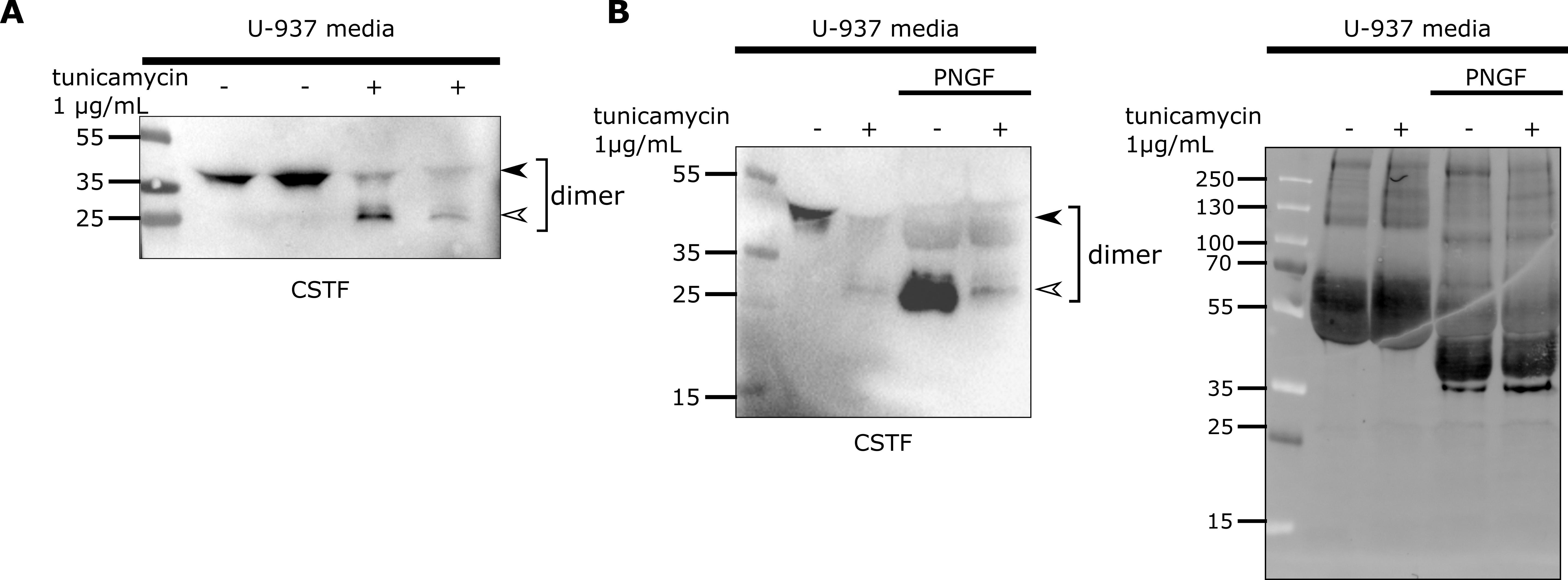


**Figure S3:** In U-937 cells treated with tunicamycin, non-glycosylated form of CST is secreted into cell media. A: Western blot showing both non-glycosylated CSTF (white arrowhead) is and fully glycosylated CSTF (black arrowhead) are secreted. B: Samples were treated with PNGF in order to show that secreted CSTF after tunicamycin treatment is indeed non-glycosylated (left), along with the stain free loading control (right).


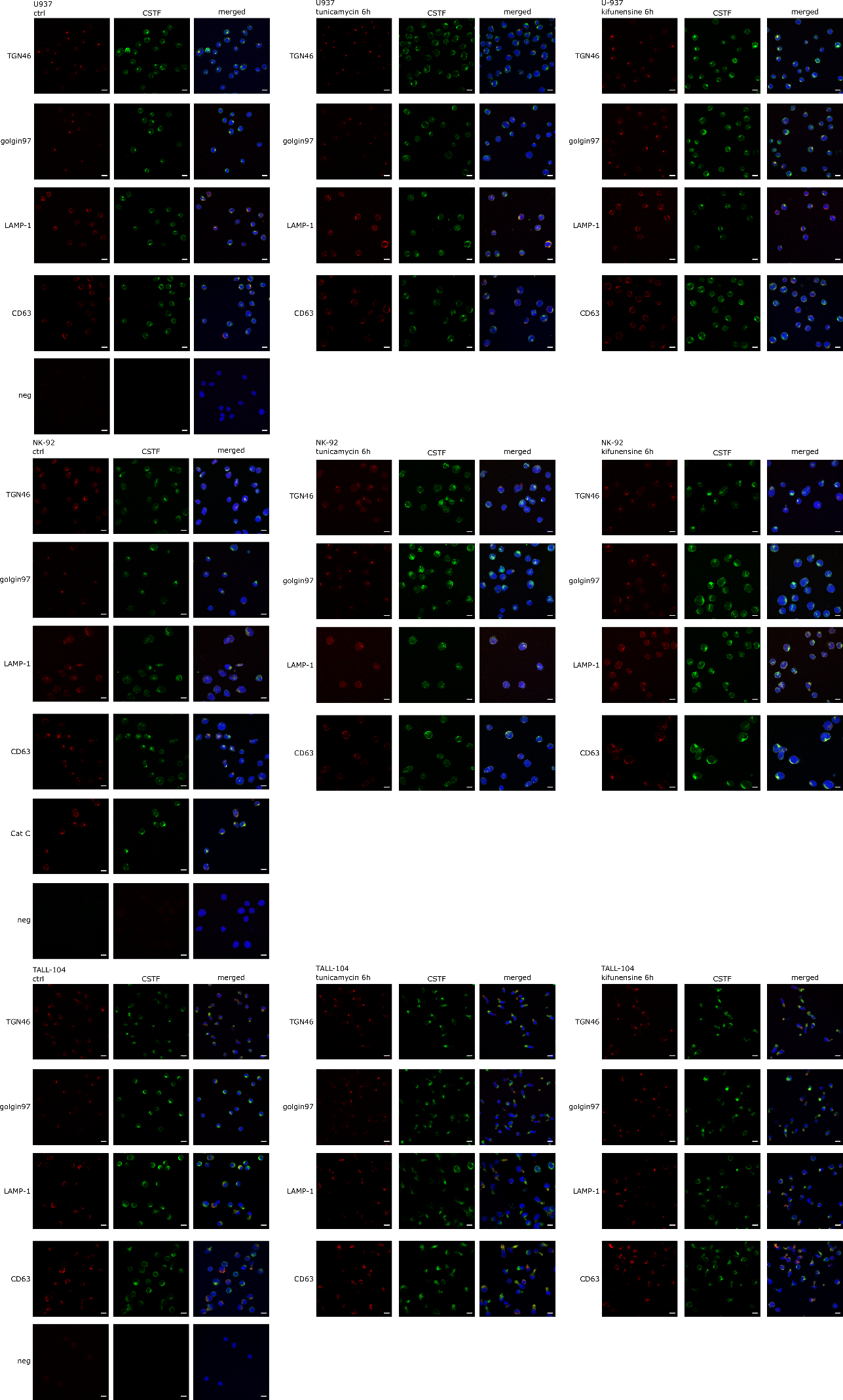


**Figure S4:** Representative confocal microscopy images of control, tunicamycin (1µg/mL, 6h) and kifunensine treated (10 µM, 6h) U-937, NK-92 and TALL-104 cells showing co-localization of CSTF (green) with TGN46, golgin-97, LAMP-1, CD63 and CatC (red) at 63x magnification. Negative staining controls were done using only secondary antibodies. Scale bars=10 µm


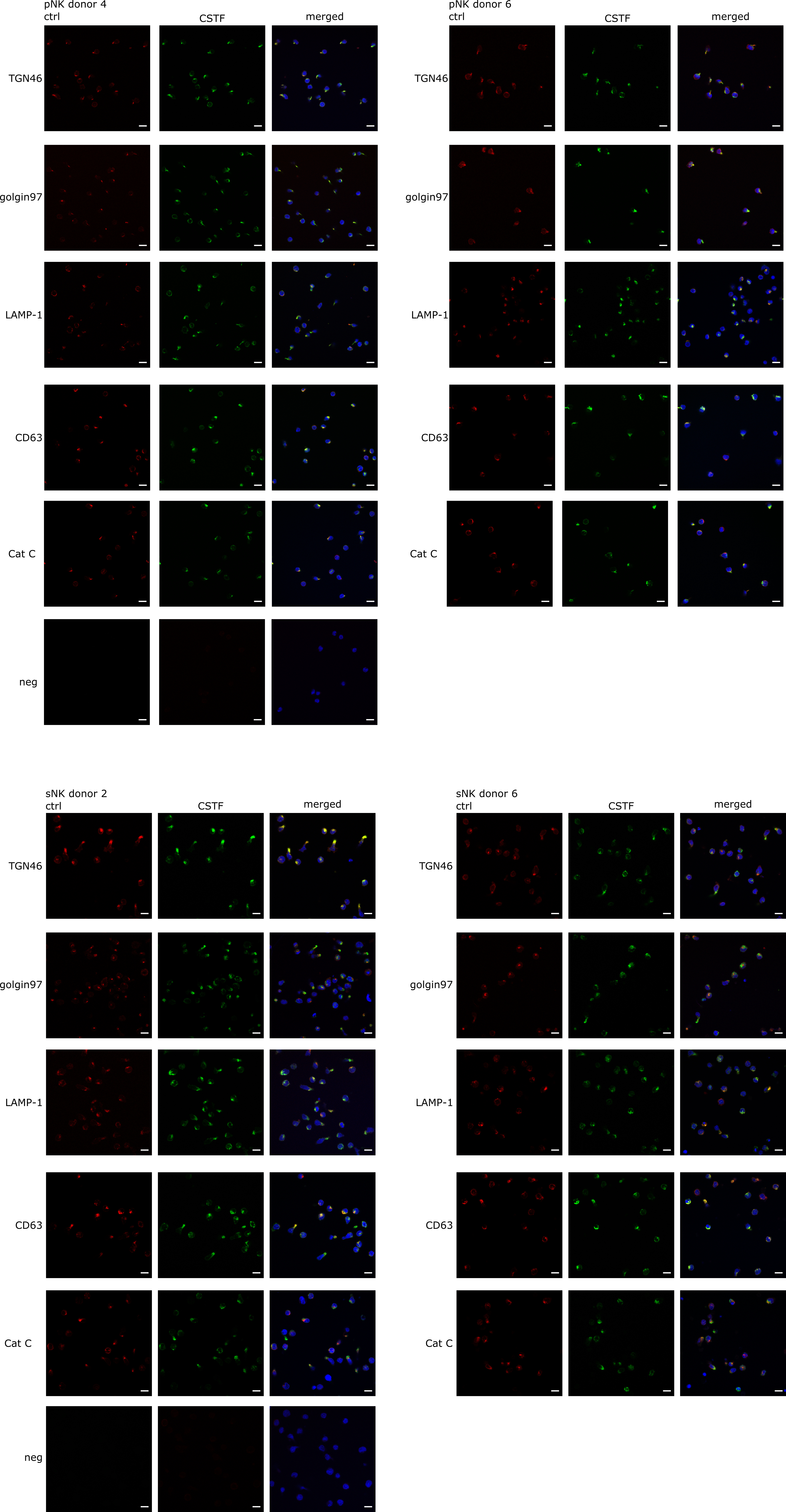


**Figure S5:** Representative confocal microscopy images of primary (pNK) and super-charged (sNK) NK cells showing colocalization of CSTF (green) with TGN46, golgin-97, LAMP-1, CD63 and CatC (red) at 63x magnification. Negative staining controls were done using only secondary antibodies. Scale bars=10 µm


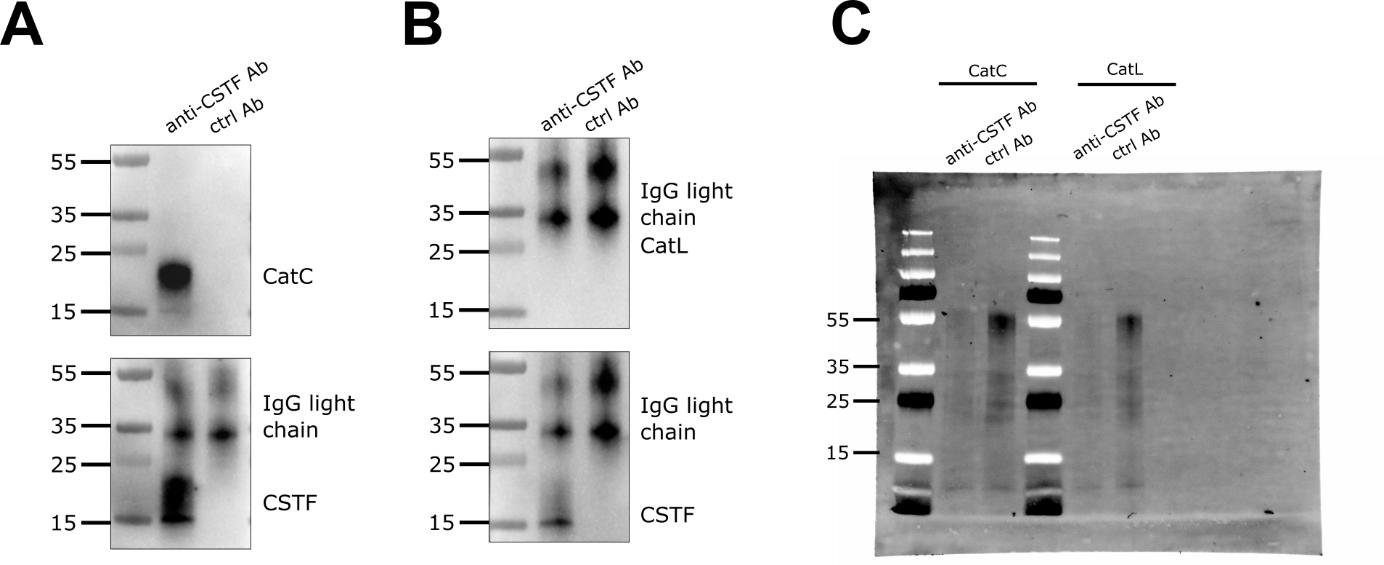


**Figure S6:** Western blot analysis of interaction of CSTF with CatC or CatL in immunoprecipitates of supercharged NK cell lysates with anti-CSTF antibodies and control antibodies against lectin isolated from Macrolepiota procera. A: Immunoblot for CatC (upper panel) and CSTF (lower panel). B: Immunoblot for CatL (upper panel) and CSTF (lower panel). C: Imaging of stain-free activated protein membrane was used to confirm protein loading.


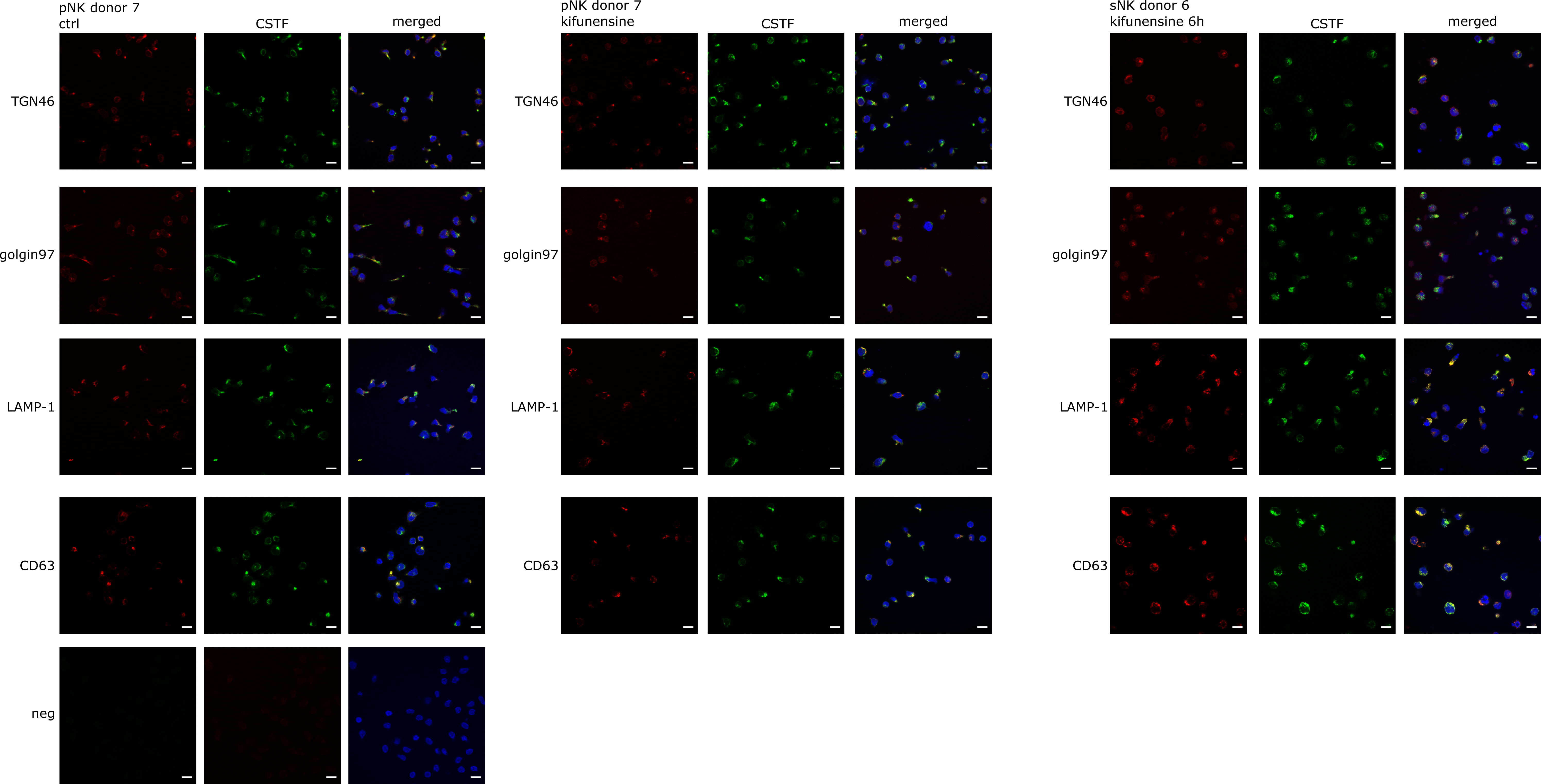


**Figure S7:** Representative confocal microscopy images of primary (pNK) and super-charged (sNK) NK cells treated with 10 µM kifunensine for 6h, showing colocalization of CSTF (green) with TGN46, golgin-97, LAMP-1, and CD63 (red) at 63c magnification.. Negative staining controls were done using only secondary antibodies. Scale bars=10 µm


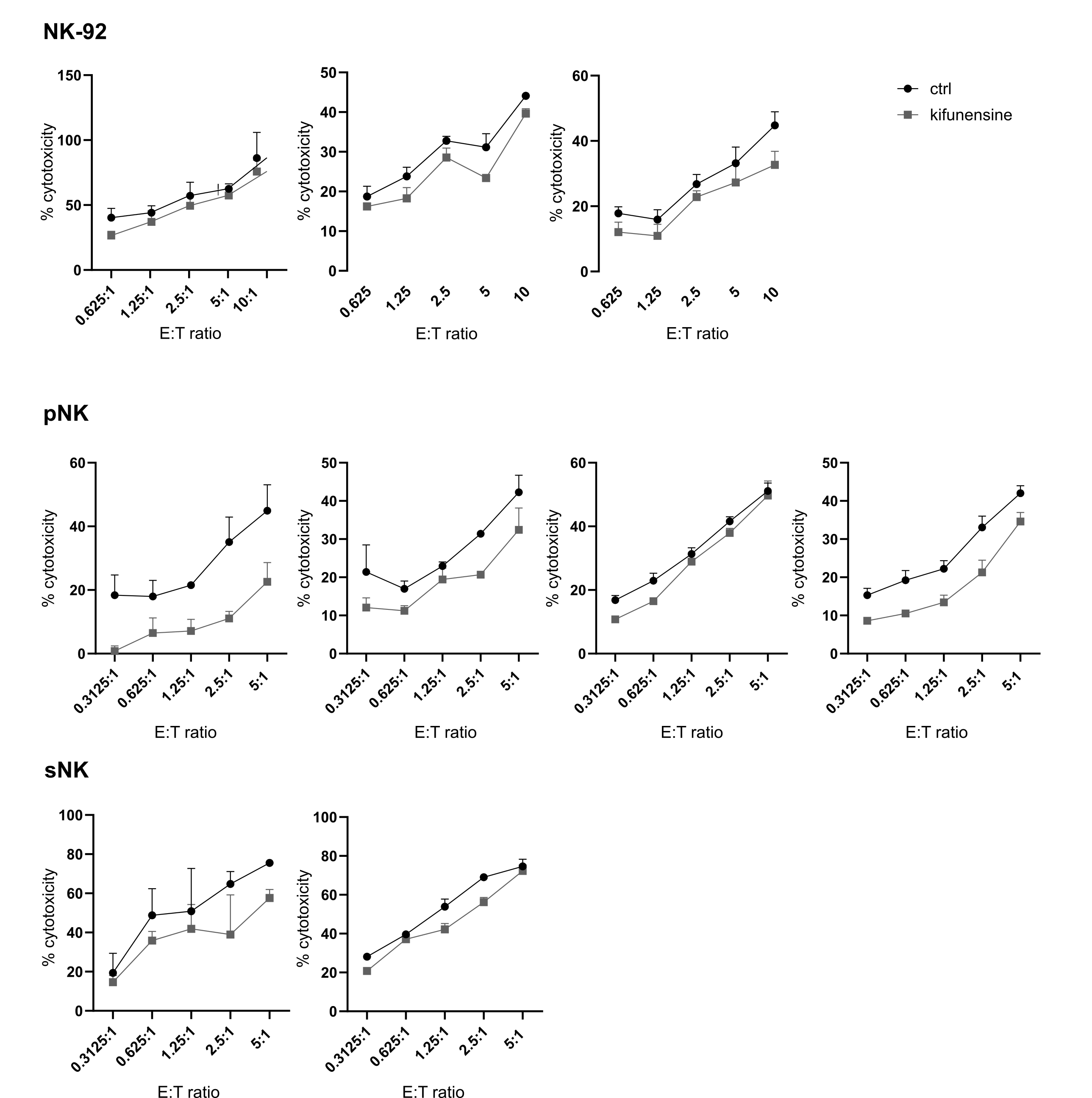


**Figure S8:** Cytotoxicity assay of NK-92 cells (upper panel), primary NK cells (middle panel) and super-charged NK cells (bottom panel) untreated or treated with 20 µM kifunensine (grey) for 6h prior to incubation with calcein-AM labelled K-562 cells. Graphs represent biological replicates for NK-92 cells or different donors for primary and super-charged NK cells. Assay was conducted in triplicates.


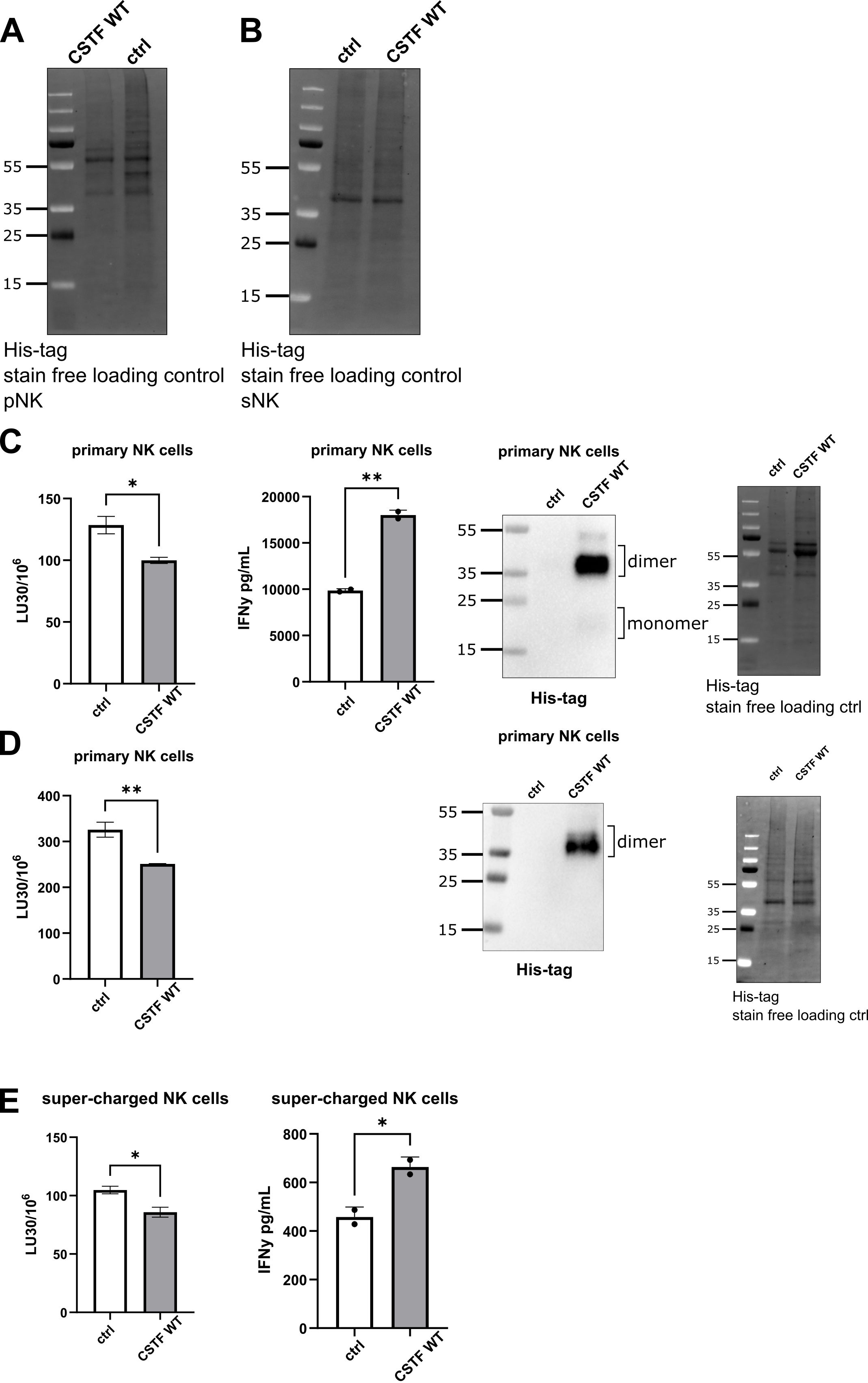


**Figure S9:** Loading control for anti-his-tag western blot for primary NK cells (pNK) (A) and super-charged NK cells (sNK) (B). Effects on cytotoxicity of pNK (C, D) and sNK cells (E) after 2h treatment with 100 nM recombinant CSTF (left). IFNγ secretion measured with ELISA after 18h treatment with 100 nM recombinant CSTF in pNK (C, D) and sNK (E) cells (middle). Western blot detection of his-tag residue of recombinant CSTF and loading controls of pNK (C, D) and sNK (E), treated with 100 nM recombinant CSTF for 18h (right). White bars represent control samples, and grey bars represent CSTF treated samples. Asterisks indicate statistically significant differences (* p<0.05, ** p<0.01).


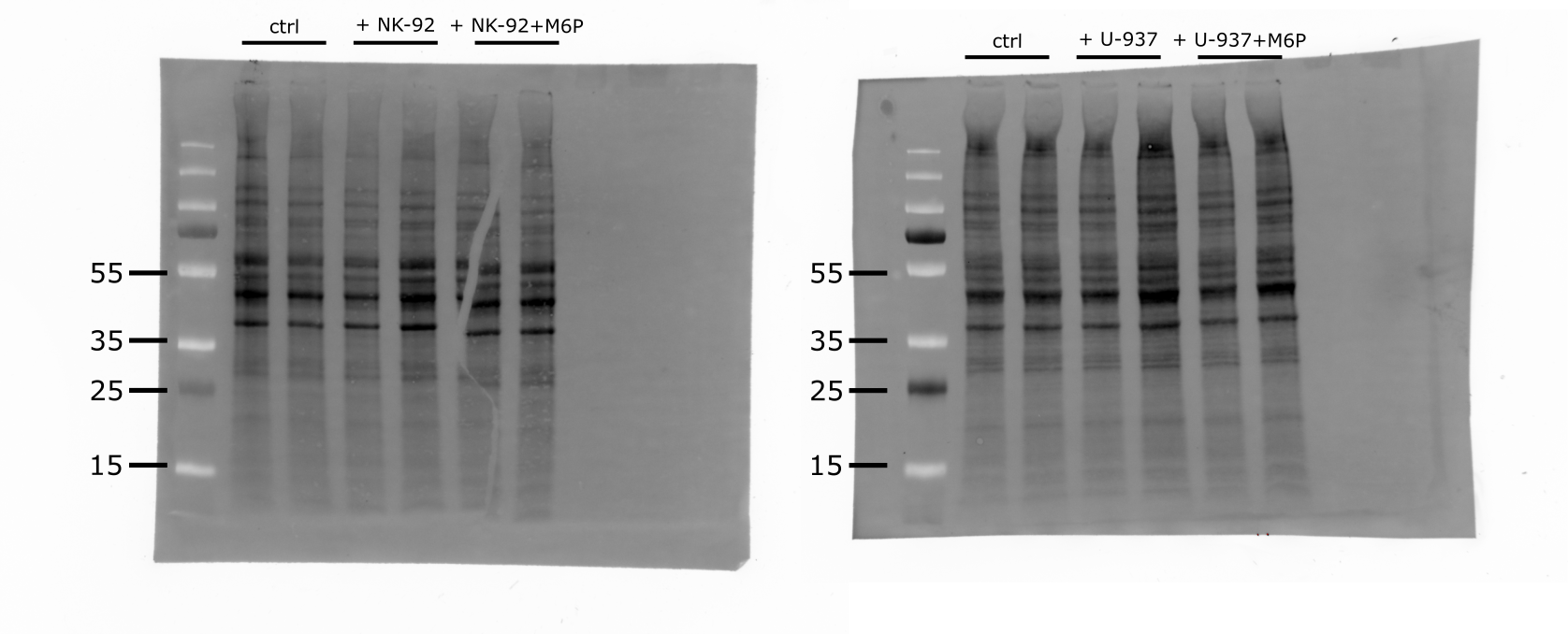


**Figure S10** Stain free loading controls for internalization of CSTF to U-251 MG cells from NK-92 conditioned media (A) and U-937 (B) conditioned media.


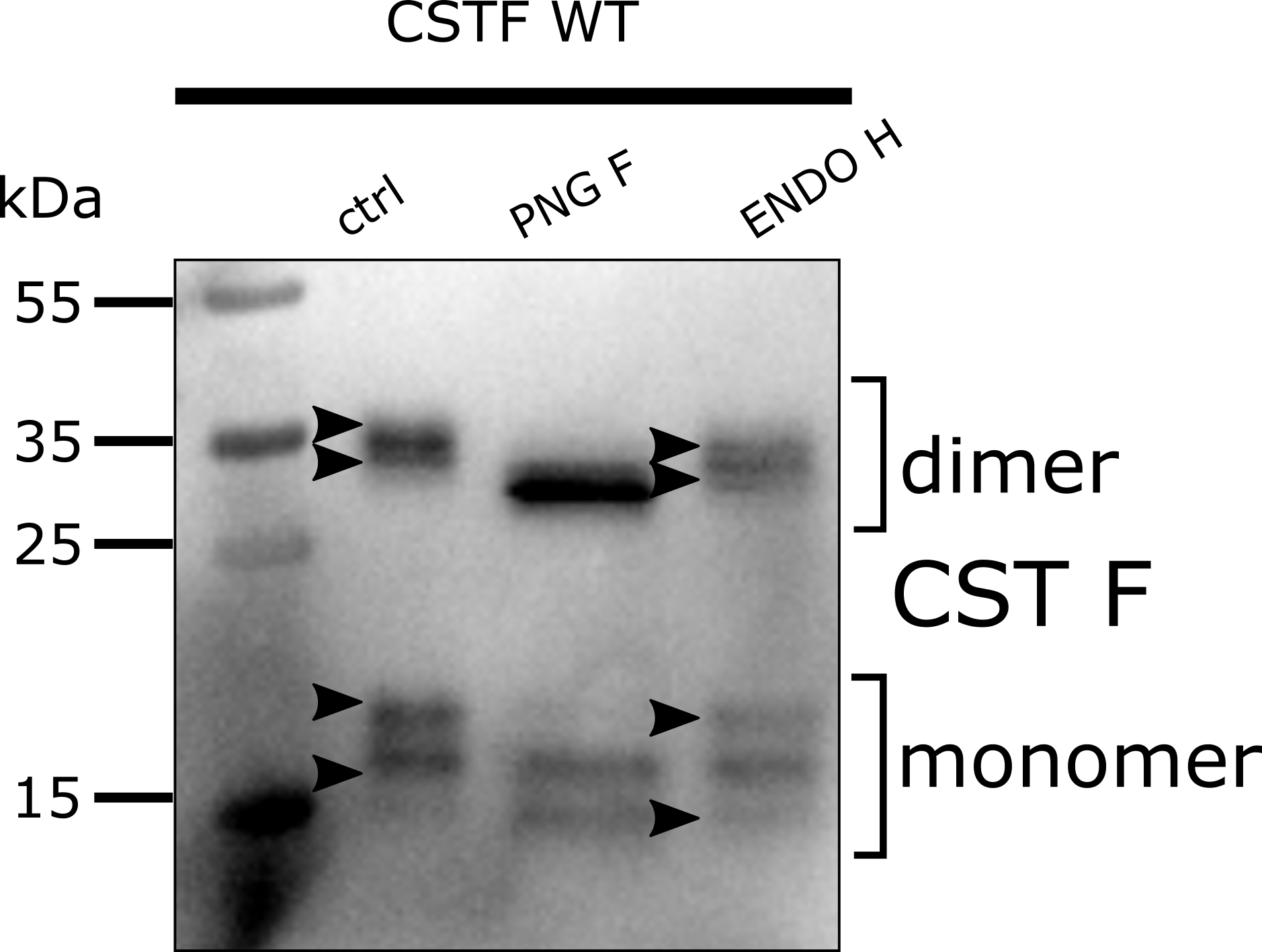


|  |  | position | CSTF WT |
| --- | --- | --- | --- |
| CSTF | **DIMER** | Asn62 | Paucimannose |
|  |  | Asn115  (predominantly glycosylated) | Paucimannose |
|  | **MONOMER** | Asn62 | Paucimannose |
|  |  | Asn115  (predominantly glycosylated) | Paucimannose |

**Figure S11:** Glycosylation of secreted cystatin F produced in HEK293F cells (CSTF WT) analysed by LC-MS/MS (left) and by western blot with deglycosylation enzymes (right).
